# Reduction of fibrosis and immune suppressive cells in ErbB2-dependent tumorigenesis by an LXR agonist

**DOI:** 10.1371/journal.pone.0248996

**Published:** 2021-03-29

**Authors:** Gao Sheng, Hongyan Yuan, Lu Jin, Suman Ranjit, Julia Panov, Xun Lu, Moshe Levi, Robert I. Glazer

**Affiliations:** 1 Department of Oncology and Lombardi Comprehensive Cancer Center, Georgetown University, Washington, DC, United States of America; 2 Department of Breast, Women’s Hospital of Nanjing Medical University, Nanjing Maternity and Child Health Care Hospital, Nanjing, China; 3 Department of Biochemistry and Molecular Biology, Georgetown University, Washington, DC, United States of America; 4 Faculty of Natural Sciences, University of Haifa, Haifa, Israel; 5 George Washington University, Washington, DC, United States of America; Université Clermont Auvergne - Faculté de Biologie, FRANCE

## Abstract

One of the central challenges for cancer therapy is the identification of factors in the tumor microenvironment that increase tumor progression and prevent immune surveillance. One such element associated with breast cancer is stromal fibrosis, a histopathologic criterion for invasive cancer and poor survival. Fibrosis is caused by inflammatory factors and remodeling of the extracellular matrix that elicit an immune tolerant microenvironment. To address the role of fibrosis in tumorigenesis, we developed NeuT/ATTAC transgenic mice expressing a constitutively active NeuT/erbB2 transgene, and an inducible, fat-directed caspase-8 fusion protein, which upon activation results in selective and partial ablation of mammary fat and its replacement with fibrotic tissue. Induction of fibrosis in NeuT/ATTAC mice led to more rapid tumor development and an inflammatory and fibrotic stromal environment. In an effort to explore therapeutic options that could reduce fibrosis and immune tolerance, mice were treated with the oxysterol liver X receptor (LXR) pan agonist, N,N-dimethyl-3-β-hydroxy-cholenamide (DMHCA), an agent known to reduce fibrosis in non-malignant diseases. DMHCA reduced tumor progression, tumor multiplicity and fibrosis, and improved immune surveillance by reducing infiltrating myeloid-derived suppressor cells and increasing CD4 and CD8 effector T cells. These effects were associated with downregulation of an LXR-dependent gene network related to reduced breast cancer survival that included Spp1, S100a9, Anxa1, Mfge8 and Cd14. These findings suggest that the use of DMHCA may be a potentially effective approach to reduce desmoplasia and immune tolerance and increase the efficacy of cancer therapy.

## Introduction

Over the past decade, it has become increasingly apparent that the cell-centric hallmarks of cancer must take into account the multi-faceted role of multiple cell types in the tumor microenvironment (TME) [1–4]. During transition from pre-invasive to invasive breast cancer, the TME undergoes extensive extracellular matrix remodeling [5] and expresses a stromal-derived gene expression signature indicative of poor outcome in multiple breast cancer subtypes [6]. In hormone receptor-negative breast cancer, the repertoire of stromal cells in the TME [4, 7, 8] produces fibrotic foci and earlier invasion [9], which elicit the secretion of inflammatory factors that contribute to immune suppression in multiple ways [4, 10, 11], including the secretion of a dense fibrotic collagen matrix that impedes the penetration of cytotoxic CD8^+^ effector T cells (CTL) into the tumor [12]. Additionally, these inflammatory factors facilitate the recruitment of regulatory T cells (Treg), myeloid-derived suppressor cells (MDSC) and tumor-activated macrophages, which collectively inhibit CTL activation and antigen presentation [13–15]. Fibrosis is also accompanied by metabolic changes, including COX2/PTGS2 activation [16], which elicits an inflammatory stress response [17, 18] as well as the suppression of CTL activation by glycolysis [19]. These outcomes suggest that therapy targeting the inflammatory and desmoplastic TME may be an effective approach to reduce immune tolerance and enhance the efficacy of cancer therapy [13, 15]. LXRs play an important role in desmoplasia by transrepression of NFκB-activated pro-inflammatory genes, including IL1, IL6, PTGS2/COX-2, MMP9 and TNF [20, 21], which accounts in part for their anti-fibrotic activity in the kidney, liver, heart, lung and retina [22–26]. To address the relationship between LXR activation and fibrosis in mammary tumorigenesis, we determined whether treatment of fibrotic NeuT/ATTAC mice [27] with the oxysterol liver X receptor (LXR) agonist, N, N-dimethyl-3-β-hydroxy-cholenamide (DMHCA) [28], could reduce fibrosis, tumor progression and immune tolerance. Our results suggest that DMHCA may be a promising therapeutic adjunct for improving the outcome of HER2/ErbB2 breast cancer.

## Materials and methods

### Animals

MMTV-NeuT/ATTAC mice [27] were derived from MMTV-NeuT mice (FVB-Tg(MMTV-Erbb2)NK1Mu/J, Jackson Labs) expressing a constitutively active rat ErbB2[V664E] gene [29, 30], and FAT-ATTAC mice expressing an FKBPv-caspase-8 fusion protein under the control of the FABP4 promoter (kindly provided by Dr. Philipp Scherer, University of Texas Southwestern) [31, 32]. Animal studies were approved by the Georgetown University Animal Care and Use Committee (GUACUC) in accordance with NIH guidelines for the ethical treatment of animals. Mice were not maintained for extended periods where tumors could cause discomfort, and analgesics were not used due to their possible interference with DMHCA bioavailability. Mice were observed daily for tumors, and when tumor volume reached 5% of body wt or appeared necrotic, mice were euthanized in accordance with the recommendations of the American Veterinary Medical Association, https://www.avma.org/sites/default/files/2020-02/Guidelines-on-Euthanasia-2020.pdf, using carbon dioxide inhalation followed by cervical dislocation as stipulated by GUACUC.

### Treatments

Mammary gland fibrosis was induced in female six-week-old NeuT/ATTAC mice by i.p. injection of 0.4 mg/kg AP20187 (MedChemExpress) dissolved in a vehicle (4% ethanol, 10% PEG-400 and 1.75% Tween-20 in water) three times per week, and are hereafter referred to as ‘NeuT/ATTAC+AP’ mice. AP20187 is a dimer analog of FK506 and serves as a selective FKBPv-caspase 8 dimerizer resulting in partial ablation of mammary fat and its replacement by fibrotic tissue [31, 32]. At eight weeks of age, NeuT/ATTAC+AP mice were administered *ad libitum* a diet (LabDiet 5053) supplemented with 0.05% (w/w) DMHCA (WuXi App Tec, China), which is equivalent to a dose of ~100 mg/kg. No weight loss or overt toxicity resulted from AP21087 or DMHCA treatment. The treatments are summarized in S1 Fig in S1 File.

### Fluorescence-activated cell sorting (FACS)

Tumor and spleen were removed and digested with collagenase D (Roche) at a ratio of 15 ml collagenase solution per 2 g of tissue for 1 hr at 37°C with shaking [33]. The cell suspension was filtered through a 70 μm strainer, washed and erythrocytes lysed before analysis of 1x10^6^ cells by FACS. Viable cells were determined with the Live/Dead Fixable Dead Cell Stain Kit (Invitrogen) and excluded from analysis, and non-specific binding was blocked with Fc antibody CD16/32 (Biolegend). Cells were sorted for CD45^+^ cells and subsequently for macrophages (F4/80^+^/MHCII^+^), G-MDSC (CD11b^+^/Gr-1^+^), M-MDSC (CD11b^+^/Ly6C^+^), dendritic cells (CD11c^+^/MHCII^+^), T cells (CD4^+^/CD8^+^), NK cells (CD45^+^/NK1.1^+^) and Treg cells (Foxp3^+^/CD25^+^ and Foxp3^+^ /PD-1^-^). Cells were analyzed for Foxp3 after fixation in 1% paraformaldehyde and permeabilization (Permeabilization Buffer, eBioscience). Analysis was conducted by the Flow Cytometry & Cell Sorting Shared Resource using a BD LSRFortessa analyzer (BD Biosciences) and FCS Express 4 software (De Novo Software). Antibodies are listed in S1 Table in S1 File.

### Immunohistochemistry

Mammary tissue was excised and FFPE sections were prepared as previously described for IHC [27, 34]. Antibodies are listed in S1 Table in S1 File. Tissues from HER2+ breast cancer subjects were deidentified and hence did not require approval by an ethics committee.

### Quantitative real-time polymerase chain reaction qRT-PCR

RNA was extracted and reverse transcribed using the Omniscript RT kit (Qiagen) as previously described [27, 34, 35]. PCR was performed in triplicate using an ABI-Prism 7700 (Applied Biosystems) and SYBRGreen I detection (Qiagen) according to the manufacturer’s protocol. Amplification using the appropriate primers was confirmed by ethidium bromide staining of the PCR products on an agarose gel. The expression of each target gene was normalized to GAPDH and is presented as the ratio of the target gene to GADPH expression calculated using the formula, 2^-ΔCt^, where ΔCt = Ct^Target^-Ct^18s^ [35]. RT-PCR primers are listed in S2 Table in S1 File.

### Second harmonic generation (SHG) and fluorescence lifetime microscopy (FLIM)

Phasor-mapped FLIM and SHG images were acquired with an Olympus FVMPE-RS (Olympus, Waltham, MA) upright microscope equipped with an Insight X3 laser (Spectra-Physics, Santa Clara, CA) and a DIVER (Deep Imaging Via Enhanced Recovery) detector [36, 37]. Samples were excited with a 740 nm laser in a two-photon excitation scheme at a laser repetition rate of 80 MHz. Samples were placed directly on the acquisition window, excited with a 10X air objective (NA-0.3, UPLFLN10X2) (Olympus, Waltham, MA), and SHG signals were collected with the DIVER detector [38, 39] at 370±10 nm with a combination of UG11 and BG39 filters. Signals were recorded with a FLIMBox (ISS, Champaign, IL) and converted to a phasor plot with SimFCS that was developed by Dr. Enrico Gratton, Laboratory for Fluorescence Dynamics, University of California at Irvine (https://www.lfd.uci.edu/globals). Phasor plots were calibrated using Rhodamine 110 in water, τ = 4.0 nsec [40]. The pixel dwell time was 20 μsec, and the images scanned 16 times to increase the signal to noise ratio of the phasor plot. The scanner was controlled by the Olympus microscope and images were collected in the passive mode with a zoom of 1 corresponding to an image size of 1.2 mm. FLIM data were analyzed graphically by phasor plots to obtain information on multiple fluorescence components [41–43]. The distribution of phasor points originating from FLIM measurements for mono-exponential and multi-exponential decays appear on or inside, respectively, the universal semicircle [42] (see Fig 4A and 4B). For SHG microscopy, there is no delay between the laser pulse and fluorescence, and therefore SHG appears at S = 0, G = 1 in the phasor plot, which distinguishes it from autofluorescence [38, 39, 42] (see Fig 4E).

### RNAseq analysis

RNA was extracted and its quality assessed as previously described [27, 33]. RNAseq was done by 10X Genomics. Raw data quality was checked using FastQC (v0.11.9), and adapter trimming on raw data was performed using Cutadapt (v2.9). The reference genome was downloaded from Ensembl mm10 release 99, and the reference genome index was built using Bowtie2 (v2.4.1) software. Paired-end trimmed reads alignment and raw read count calculation were performed using RSEM software (v1.3.1). Statistical analysis were performed using the DESeq2 package (v1.26) in R (v3.6). Genes with q-value <0.05 were considered as differentially expressed and used as input for Gene Set Enrichment Analysis (GSEA) (v3.0, Broad Institute). RNAseq data have been deposited in the GEO database under accession no. GSE166864; https://www.ncbi.nlm.nih.gov/geo/query/acc.cgi?acc=GSE166864.

### Statistical analysis

Statistical significance of means±S.E. were evaluated using the two-tailed Student’s t test at a significance of *P*<0.05. Differences in tumor growth were determined by the log rank Mantel-Cox test at a significance of P<0.05 using Prism GraphPad software.

## Results

**Fig 1:**
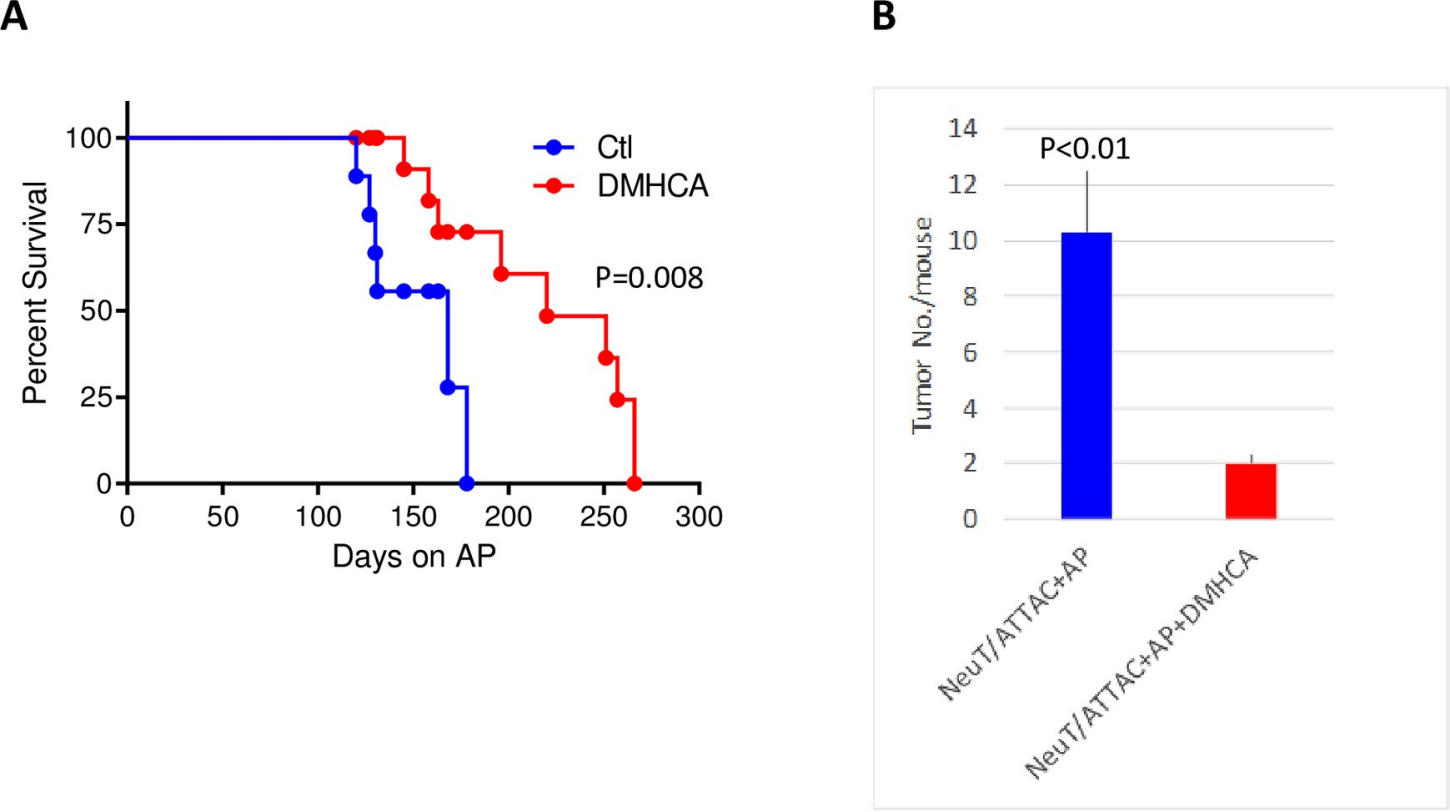
DMHCA reduced tumor progression and tumor multiplicity. Fibrosis was induced by i.p. injection of 0.4 mg/kg AP three times weekly beginning at 6 weeks of age and throughout the interval noted (‘Days on AP’). Beginning at 8 weeks of age, animals were administered a diet containing 0.05% DMHCA (100 mg/kg) until tumors appeared. **A**, Survival analysis of tumor progression following DMHCA treatment of NeuT/ATTAC+AP mice. Statistical significance was determined by the log rank Mantel-Cox test. **B**, Tumor multiplicity following DMHCA treatment. Statistical significance was determined by the two-tailed t test.

The LXR pan agonist DMHCA reduces fibrosis in several non-tumorigenic disease models [23, 38, 44, 45], and therefore we evaluated its efficacy in our conditional NeuT/ATTAC+AP mammary fibrosis model expressing a constitutively active ErbB2 gene and LXRβ/NR1H2 as the major isoform [27]. In this transgenic model, forced dimerization of the fat-directed FKBPv-caspase transgene by dimerizer AP21087 results in partial ablation of mammary fat, but not visceral fat [31, 32], and its replacement with fibrotic tissue [31, 32]. Continuous treatment of two month-old NeuT/ATTAC+AP mice with a diet containing 0.05% DMHCA over several months significantly increased survival (Fig 1A) and reduced tumor multiplicity by four-fold (Fig 1B).

**Fig 2:**
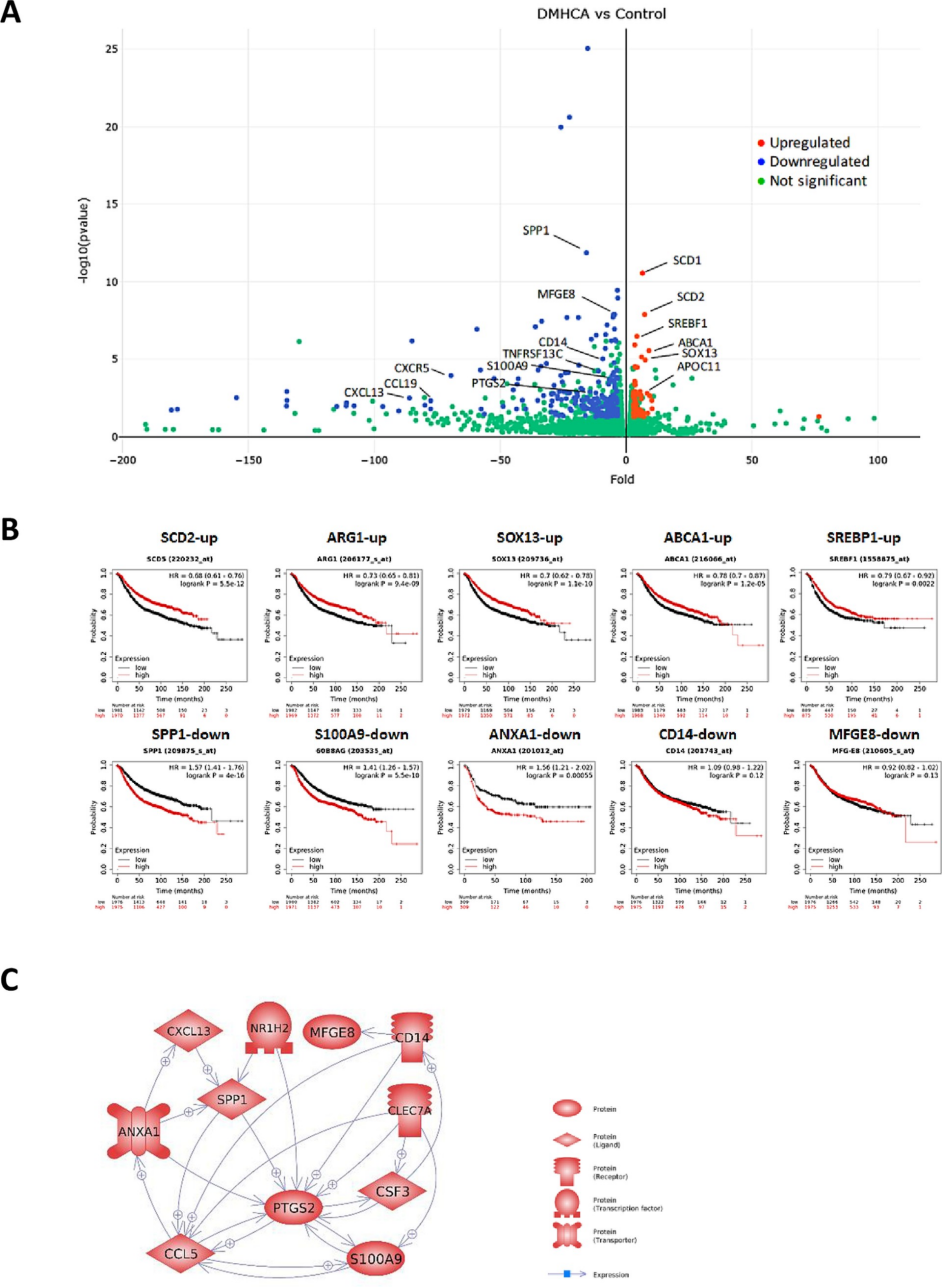
RNAseq analysis of tumors from NeuT/ATTAC+AP mice treated with DMHCA. **A**, Volcano plot comparing up- and downregulated genes with a p-value <0.05 and a ≥3-fold change. **B**, Kaplan-Meier analysis of survival probability in all breast cancer subjects with high or low expression of LXR-modulated genes. **C**, The interrelationship of LXR-modulated genes downregulated in tumors from NeuT/ATTAC+AP mice after DMHCA treatment.

To provide context for the inhibitory effects of DMHCA, tumors from three control mice and three DMHCA-treated mice were analyzed by RNAseq. Although this few number of tumors might be considered a limitation, only genes with a raw score ≥300 representing a ≥3-fold change with a p-value adjusted for multiple comparisons (padj) of <0.05 were evaluated for functional significance (Tables 1–4 and S3 Table in S1 File). RNAseq analysis revealed statistically significant changes in 289 genes, of which 78 were upregulated and 211 downregulated by DMHCA treatment (Fig 2A and S3 Table in S1 File). Approximately 14% of the upregulated and 6% of the downregulated genes contained an LXR response element (LXRE) [46], and many of the downregulated genes are known to be enriched in malignancies, fibrosis, immune cell infiltration and inflammatory disorders (Table 1). There was close agreement between the results of RNAseq and qRT-PCR for the downregulated genes in Fig 2C (S2 Fig in S1 File). Several of the LXRE genes predicted increased regression-free survival in breast cancer subjects (Table 2 and Fig 2B), among which Ptgs2, Mfge8, Anxa1, Spp1, S100a9 and Cd14 are biomarkers for MDSC and Treg cells (Table 3). Interestingly, many of the DMHCA-modulated genes are classified as damage-associated molecular patterns (DAMPS) known to promote pathological inflammatory responses [47] (Table 4).

**Table 1:** Disease networks enriched in genes downregulated in tumors from NeuT/ATTAC+AP mice treated with DMHCA.

Disease	Overlap	Overlapping Entities	p-value	Jaccard similarity
**neutrophil infiltration**	22	**CD14;ANXA1;PTGS2,S100A9;MFGE8;SPP1;CXCL13;CLEC7A;CSF3;CCL5;**ENPP2;ADORA1;MMP12;SFTPD;ANGPT1;SLPI;	1.94E-16	2.81E-02
		SLC12A2;MMP3;PTGER4;PTN;SELL;NPY		
**chronic inflammation**	23	**CD14;PTGS2;S100A9;MFGE8;SPP1;CXCL13;CSF3,CCL5**;MMP12;PTGES;AURKA;SPARCL1;ENPP2;AREG;SFTPD;ANGPT1;	2.29E-15	2.34E-02
		ARG1;LTB;MMP3;PTGER4;SELL;NPY		
**inflammatory disease**	25	**CD14;ANXA1;PTGS2;S100A9;MFGE8;SPP1;CSF3;CCL5;CXCL13;CLEC7A;**ENPP2;SFTPD;CD83;SLPI;PTGES;MMP3;PTGER4;	1.46E-14	1.90E-02
		WNT7B;ADORA1;MMP12;AREG;ANGPT1;ARG1;SELL;NPY		
**leukocyte infiltration**	20	**CD14;ANXA1;PTGS2,S100A9;SPP1;CSF3;CCL5**;MMP12;CSN2;ENPP2;AREG;SFTPD;ANGPT1;CD83;SLPI;PTGES;LTB;	8.56E-14	2.44E-02
		MMP3;PTGER4;SELL		
**fibrosis**	31	**CD14;ANXA1;PTGS2,S100A9;MFGE8;SPP1;CSF3;CCL5;CLEC7A**;AURKA;SPARCL1;ENPP2;SFTPD;SLPI;PTGES;SREBF1;	1.10E-13	1.31E-02
		ADAM33;MMP3;PTGER4;LOXL4;WFDC2;SMOC1;ADORA1;MMP12;AREG;ANGPT1;ARG1;PDGFD;PTN;SELL;NPY		
**metastasis**	46	**ANXA1;PTGS2;S100A9;MFGE8;SPP1;CSF3;CCL5;CXCL13;CLEC7A;**PIR;PARD3B;FAM20C;AURKA;SPARCL1;ENPP2;SEMA3B;	1.34E-13	8.08E-03
		SOX13;KRT7;SLPI;PTGES;SLC12A2;SREBF1;AJAP1;MMP3;PTGER4;TYRO3;LOXL4;WNT7B;DEPTOR;LEF1;WFDC2;S100A6;		
		MMP12;SEMA3D;AREG;ANGPT1;S100A14;PIK3R5;STARD13;ARG1;LTB;PDGFD;PKP1;PTN;SELL;NPY		
**macrophage infiltration**	20	**CD14;ANXA1;PTGS2;S100A9;SPP1;CLEC7A;CSF3;CCL5;**;ADORA1;MMP12;ENPP2;SFTPD;ANGPT1;ARG1;SREBF1;PDGFD;	5.52E-13	2.22E-02
		MMP3;PTGER4;LOXL4;NPY		
**inflammation**	42	**CD14;ANXA1;PTGS2;S100A9;MFGE8;SPP1;CSF3;CXCL13;**;PIR;AURKA;SPARCL1;ENPP2;SFTPD;SOX13;CD83;SLPI;PTGES;	3.53E-12	8.23E-03
		SLC12A2;SREBF1;ADAM33;MMP3;PTGER4;TYRO3;DEPTOR;CCL5;WFDC2;SMOC1;ADORA1;S100A6;MMP12;CSN2;AREG;		
		IL18R1;ANGPT1;MAL;ARG1;LTB;PDGFD;PTN;SELL;NPY		
**neoplasm**	49	**CD14;ANXA1;PTGS2;S100A9;MFGE8;SPP1;CXCL13;CSF3;CCL5;CLEC7A;**;NEXMIF;AURKA;KRT7;SLC12A2;SREBF1;PTGER4;	3.93E-11	6.53E-03
		LOXL4;DEPTOR;LEF1;ADORA1;AREG;MAL;STARD13;ARG1;LTB;PDGFD;PKP1;PTN;CD37;SPARCL1;ENPP2;SEMA3B;SFTPD;		
		CD83;SLPI;PTGES;ADAM33;AJAP1;MMP3;TYRO3;WNT7B;SMOC1;MMP12;SEMA3D;IL18R1;ANGPT1;S100A14;SELL;NPY		
**neutrophil accumulation**	12	**CD14;ANXA1;PTGS2;S100A9;SPP1;CLEC7A;CSF3;CCL5;**ADORA1;AREG;SLPI;SELL	6.74E-10	2.97E-02
**lung metastasis**	20	**ANXA1;PTGS2;S100A9;SPP1;CSF3;CCL5;**MMP12;AURKA;ENPP2;SFTPD;S100A14;KRT7;PTGES;AJAP1;PDGFD;MMP3;	1.78E-09	1.44E-02
		LOXL4;SELL;WNT7B;LEF1		
**adenocarcinoma**	15	**ANXA1;PTGS2;S100A9;CSF3;**MMP12;AURKA;ANGPT1;S100A14;MAL;LTB;MMP3;PTGER4;WNT7B;LEF1;WFDC2	2.85E-09	1.98E-02
**neoplasm invasion**	20	**ANXA1;PTGS2;S100A9;SPP1;CCL5**;MMP12;ENPP2;AURKA;AREG;ANGPT1;SLPI;PIK3R5;SLC12A2;CSF3;PDGFD;MMP3;	7.88E-09	1.32E-02
		LOXL4;PTN;SELL;LEF1		
**breast cancer**	31	**CD14;ANXA1;PTGS2;S100A9;SPP1;MFGE8;CSF3;CCL5;CLEC7A;**ENPP2;SEMA3B;KRT7;AURKA;SPARCL1;SREBF1;AJAP1;	9.12E-09	8.54E-03
		MMP3;PTGER4;TYRO3;WNT7B;ADORA1;MMP12;AREG;ANGPT1;S100A14;PIK3R5;STARD13;ARG1;PDGFD;PKP1;PTN		
**cancer**	41	**CD14;ANXA1;PTGS2;S100A9;MFGE8;SPP1;CSF3;CCL5;CXCL13;**AURKA;CD37;SPARCL1;ANXA1;ENPP2;SEMA3B;CD83;	2.11E-08	6.51E-03
		KRT7;SLPI;PTGES;SREBF1;MMP3;PTGER4;TYRO3;LOXL4;SGSM1;WNT7B;DEPTOR;LEF1;WFDC2;SMOC1;S100A6;MMP12;		
		SEMA3D;AREG;ANGPT1;S100A14;MAL;PIK3R5;PDGFD;PTN;SELL;NPY		
**autoimmunity**	17	**CD14;ANXA1;PTGS2;S100A9;MFGE8;SPP1;CXCL13;CSF3;CLEC7A;CCL5**;MMP12;SFTPD;CD83;LTB;TYRO3;SELL;NPY	8.97E-08	1.35E-02

Genes are from the RNAseq results in S3 Table in S1 File. Genes depicted in Fig 2C containing an LXR response element are in **BOLD**. Shown are groups containing ≥12 genes with a p-value <0.05.

**Table 2:** Genes modulated by DMHCA that positively correlate with increased progression-free survival in breast cancer subjects.

Gene Symbol	Gene Name	FC	padj	Ctl Mean	DMHCA Mean
Arg1	Arginase 1	10.3	1.54E-02	1,489	15,384
Nrg1	Neuregulin 1	5.4	2.71E-02	249	1,333
**Scd2**	Stearoyl-CoA Desaturase	4.4	3.37E-05	21,644	95,956
**Srebf1**	Serum response element binding protein 1	4.3	3.35E-07	23,854	102,661
Arfgef2	ADP Ribosylation Factor Guanine Nucleotide Exchange Factor 2	3.8	2.28E-02	2,447	9,221
St3gal5	ST3 Beta-Galactoside Alpha-2,3-Sialyltransferase 5	3.5	3.09E-05	2,739	9,673
Tnfrsf19	TNF Receptor Superfamily Member 19	3.2	6.71E-02	867	2,817
Stard13	StAR Related Lipid Transfer Domain Containing 13	3.2	6.86E-03	674	2,184
**Lhpp**	Phospholysine Phosphohistidine Inorganic Pyrophosphate Phosphatase	3.2	3.39E-03	343	1,098
Eng	Endoglin	3.2	4.13E-02	1,584	5,060
**Bckdha**	Branched Chain Keto Acid Dehydrogenase E1 Subunit Alpha	3.2	1.21E-03	4,328	13,821
**Cpd**	Carboxypeptidase	3.1	1.28E-02	27,885	85,878
**Sox13**	SRY-Box Transcription Factor 13	3.0	1.46E-03	1,289	3,808
**Abca1**	ATP Binding Cassette Subfamily A Member 1	2.8	2.45E-03	4,862	13,428
Wnt5a	Wnt Family Member 5A	-3.1	6.59E-02	1,660	533
**S100a9**	S100 Calcium Binding Protein A9	-3.3	3.46E-02	303	87
**Cd14**	CD14Molecule	-3.6	5.34E-05	17,094	4,758
S100a6	S100 Calcium Binding Protein A6	-5.8	6.54E-07	15,295	2,619
CD209b	Dendritic Cell-Specific Intracellular Adhesion Molecules	-9.4	3.06E-03	743	80
Lyz1	Lysozyme	-9.8	4.45E-02	1,348	138
**Spp1**	Secreted Phosphoprotein 1	-15.7	1.33E-12	624,507	39,662

Tumors from NeuT/ATTAC+AP mice were analyzed by RNAseq (see S3 Table in S1 File) and include genes with a raw score >300, ≥3-fold change in expression and a padj <0.05. Genes in bold contain an LXRE response element.

**Table 3:** Genes related to immune suppression that are negatively regulated by DMHCA in tumors from NeuT/ATTAC+AP mice.

Gene Symbol	Gene Name	Fold Change	padj	Immune Cell
**S100a9**	S100 Calcium Binding Protein A9	-3.3	3.46E-02	M-MDSC
**Anxa1**	Annexin A1	-3.3	1.73E-04	M-MDSC
**Cd14**	Myeloid Cell-Specific Leucine-Rich Glycoprotein	-3.6	5.34E-05	MDSC
Clec7a	C-Type Lectin Domain Containing 7A	-3.9	1.72E-02	MDSC
Ptgs2	Prostaglandin-Endoperoxide Synthase 2	-4.4	2.95E-02	MDSC
Mfge8	Milk Fat Globule EGF And Factor V/VIII Domain Containing	-5.0	1.31E-08	Treg
Wfdc2	WAP Four-Disulfide Core Domain 2	-7.5	6.17E-08	MDSC
**Ccl5**	C-C Motif Chemokine Ligand 5	-8.9	4.21E-02	MDSC
Lyz	Lysozyme	-9.8	4.45E-02	M-MDSC
**Spp1**	Secreted Phosphoprotein 1	-15.7	1.33E-12	G-MDSC
Csf3	Colony Stimulating Factor 3	-20.7	3.31E-03	M-MDSC
Cxcl13	C-X-C Motif Chemokine Ligand 13	-24.9	4.54E-02	MDSC
Sell	Selectin L	-48.8	1.08E-02	M-MDSC

The immune suppressive cell type is based on the Human Protein Atlas (https://www.proteinatlas.org) and UniProt (https://www.uniprot.org/uniprot) databases. Genes with a padj <0.05 are listed and those containing an LXRE are in **bold**. Data are taken from S3 Table in S1 File.

**Table 4:** Damage-associated molecular patterns downregulated by DMHCA in tumors from NeuT/ATTAC+AP mice.

Gene Symbol	Gene Name	FC	padj	Ctl Mean	DMHCA Mean
**PRR Ligands**					
S100A14	S100 Calcium Binding Protein A14	-8.1	4.92E-02	1,119	139
S100A6	S100 Calcium Binding Protein A6	-5.8	6.54E-07	15,295	2,619
HMGN3	High Mobility Group Nucleosomal Binding Domain 3	-4.8	2.00E-03	1,814	377
PTGS2	Prostaglandin-Endoperoxide Synthase 2	-4.4	2.95E-02	635	144
**PTGES**	Prostaglandin E Synthase	-3.9	7.14E-02	843	218
HSPB8	Heat Shock Protein Family B (Small) Member 8	-3.5	4.21E-02	2,216	630
**S100A9**	S100 Calcium Binding Protein A9	-3.3	3.46E-02	302	87
**ANXA1**	Annexin A1	-3.3	6.82E-02	16,053	4,153
**PRRs**					
CD209B	C-Type Lectin Domain Family 4 Member L	-9.4	3.06E-03	743	80
CLEC7A	C-Type Lectin Domain Containing 7A	-3.9	1.72E-02	658	171

Shown are genes from S3 Table in S1 File with a raw score >300, ≥3-fold change in expression and a padj <0.05. Genes in **bold** contain an LXR response element. PRRs, pattern recognition receptors.

**Fig 3:**
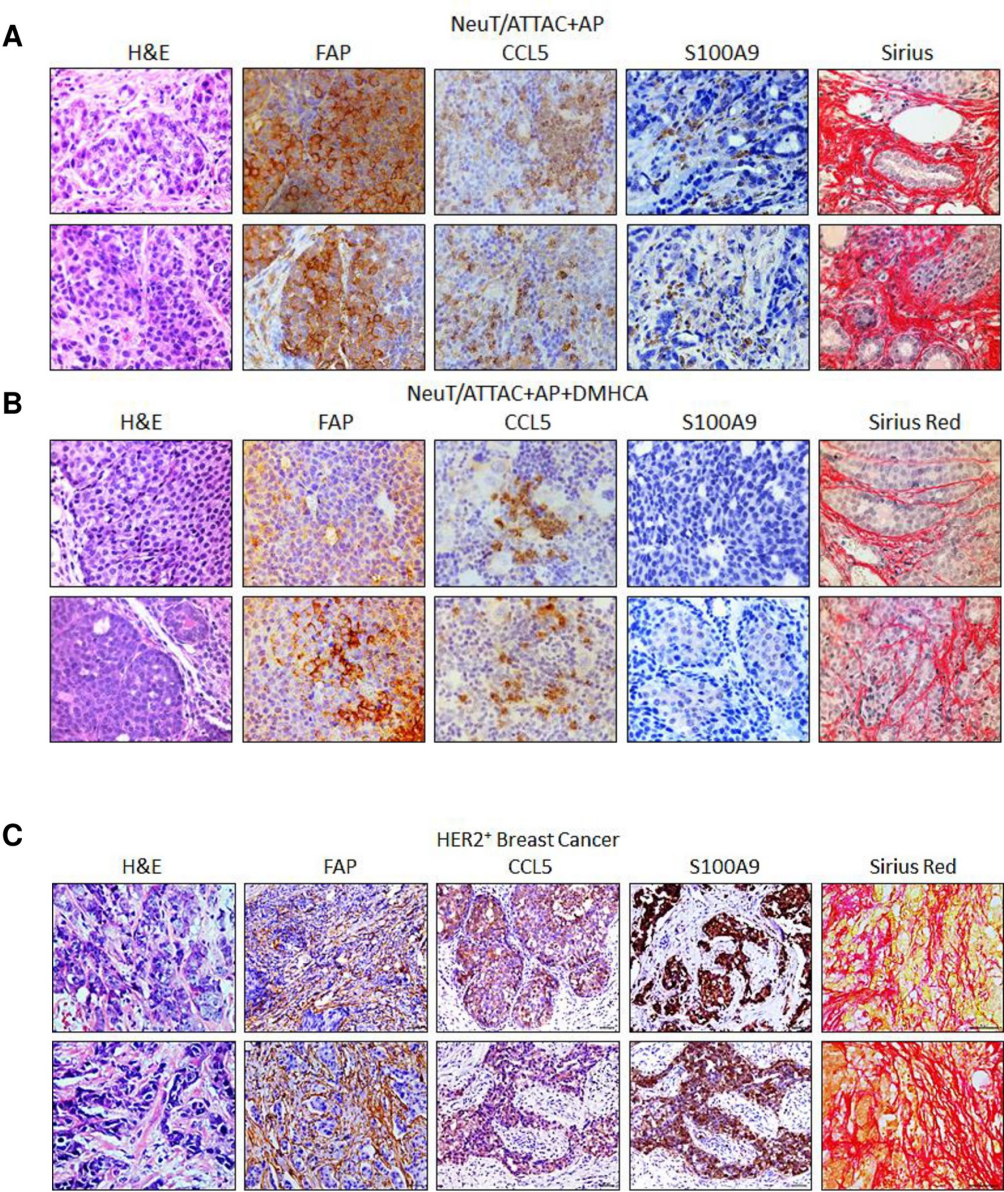
Comparison of FAP, CCL5, S100A9 and collagen expression in HER2+ breast cancer and in tumors from NeuT/ATTC+AP mice and following DMHCA treatment. NeuT/ATTAC mice were administered AP20187 and DMHCA as in Fig 1 and tissues assessed by H&E staining, FAP, CCL5 and S100A9 by IHC and collagen by PicroSirius Red staining.

**Fig 4:**
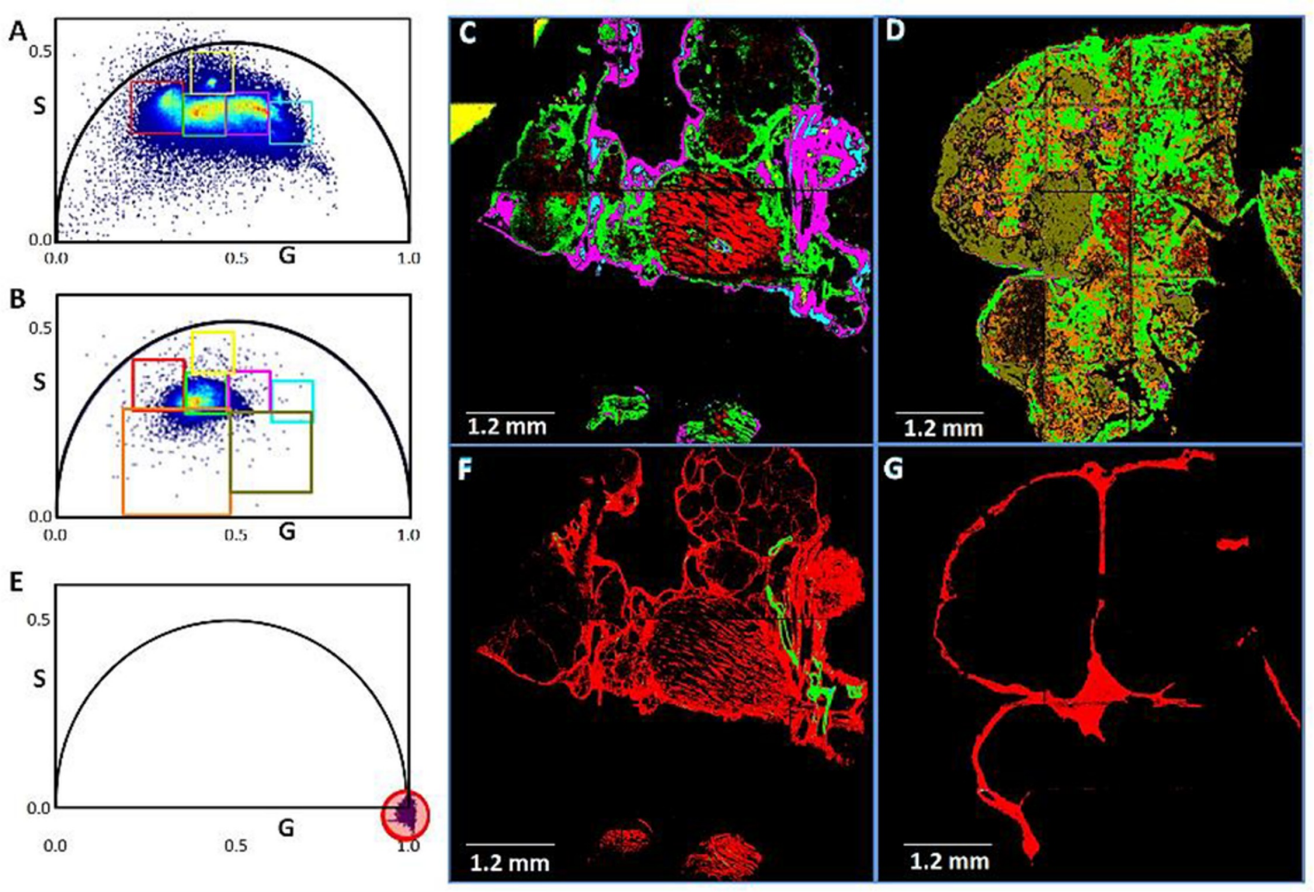
FLIM and SHG analysis of mammary tumors from control and DMHCA-treated NeuT/ATTAC mice receiving AP21087. FLIM: The red cursor shows the collagen I phasor signature, where the control tumor (**A**) has a larger spread of phasor points compared to the tumor from the DMHCA-treated animal (**B**), and indicates tumor heterogeneity. Multiple cursors selected areas of the phasor clouds for the control tumor (**A**) DMHCA-treated tumor (**B**). The control tumor shows excessive collagen I deposition shown by the red shading (**C**), as well as pink and cyan shading, which are largely absent in the DMHCA-treated tumor (**D**). The orange and olive green shading in the DMHCA-treated tumor (**D**) emphasize the changes occurring in the TME as a result of DMHCA treatment. SHG: SHG is generated from the interaction of light with the non-centrosymmetric structure of collagen I fibers (red shading), and indicates fibrosis. **E**, The phasor signature of SHG (red shaded cursor) is separated from fluorescence and appears at G = 1, S = 0), since the harmonic generation signal is not delayed compared to fluorescence, and the phasor from fluorescence appears inside the universal black semi-circle. Extensive collagen deposition is present in the control tumor (**F**) and is largely absent in the DMHCA-treated tumor (**G**), indicating a marked reduction in fibrosis. The areas represented in **F** and **G** are the same areas shown in **C** and **D**, respectively. Scale bar, 1.2 mm.

Comparison of tumors from NeuT/ATTAC+AP mice with biopsies of HER2^+^ breast cancer for fibrosis markers indicated several commonalities, including FAP (fibroblast activation protein), Ccl5, S100aA9 and collagen expression (Fig 3). Both human and murine tumors exhibited similar patterns (Fig 3A and 3B), and DMHCA treatment reduced fibrosis and expression of these biomarkers (Fig 3C). To further characterize the link between fibrosis and tumor progression, we applied the combinatorial approach of FLIM and SHG microscopy [48] used previously to analyze non-malignant fibrotic tissues [38, 39, 45, 49] (Fig 4). FLIM determines the spatial distribution of fluorescence decay at each pixel of an image to measure the cellular environment by its autofluorescence, and when used with phasor analysis provides a 2-D representation of the abundance of collagen present [50, 51]. The phasor plot of tumor tissue from control mice (Fig 4A) showed a greater spread of phasor points compared to the tumor following DMHCA treatment (Fig 4B) that is indicative of tumor heterogeneity. The FLIM image of the control tumor showed an abundance of collagen I (*green*) & collagen III (*dark red*) (Fig 4C), which were largely absent after DMHCA treatment (Fig 4D). The phasor signature of SHG microscopy (Fig 4E, *red*) indicated separation from fluorescence at G = 1, S = 0 within the universal semi-circle (*black*), and the SHG image showed an abundance of collagen I and III in the control tumor (Fig 4F), whereas markedly less collagen was present after DMHCA treatment (Fig 4G).

**Fig 5:**
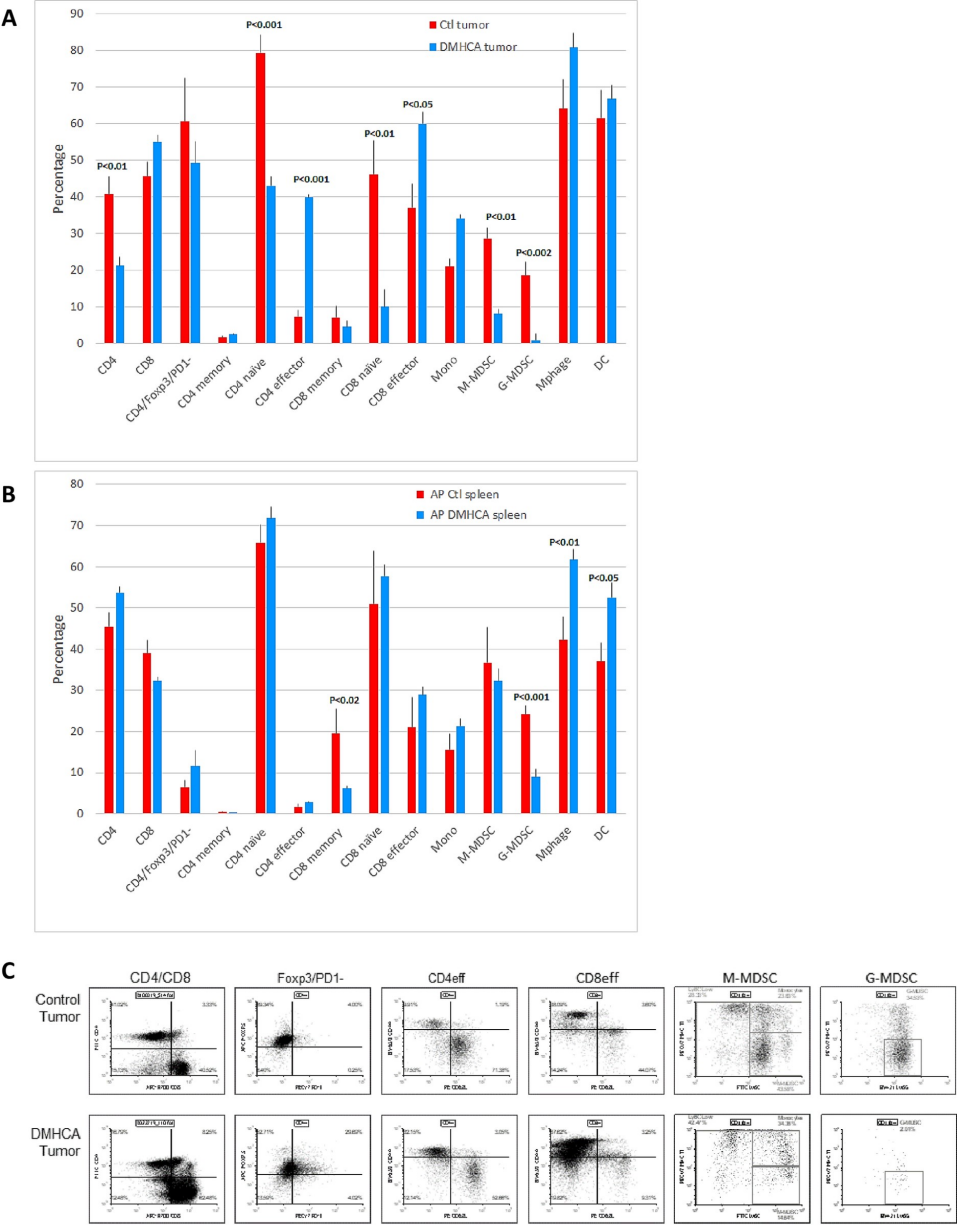
DMHCA reduces tumor infiltrating MDSC and increases CD4^+^ effector T cells. **A**, Flow cytometry analysis of immune cell subsets from tumor infiltrates and spleen after DMHCA treatment. There was a significant increase in CD4 effector T cells and a reduction in M-MDSC and G-MDSC in tumors as well as a reduction of G-MDSC in the spleen. N = 5 per group. G, granulocytic; M, monocytic. Statistical significance was determined by the two-tailed Student’s t test. **B**, Representative FACS analyses of tumor infiltrates from control and DMHCA-treated mice.

We then determined whether DMHCA treatment resulted in changes in the immune TME of NeuT/ATTAC+AP mice. DMHCA produced a significant increase in CD4 and CD8 effector T cells (CD44^+^/CD62L^-^) (Fig 5A and 5B) as well as a reduction in both naïve (CD44^-^/CD62L^+^) T cell populations (Fig 5A). Although DMHCA did not reduce the primary population of circulating Treg cells (CD4^+^/Foxp3^+^/PD-1^-^) [52], it did reduce the percentages of both M-MDSC and G-MDSC in tumor infiltrates (Fig 5A) and G-MDSC in the spleen (Fig 5B) and increased the percentages of macrophages and dendritic cells in the spleen (Fig 5B), but not in the tumor.

## Discussion

The present study has assessed the role of the LXR agonist DMHCA in reducing tumorigenesis and ameliorating fibrosis and immune tolerance in the NeuT/ATTAC fibrosis model of ErbB2 neoplasia [27]. DMHCA was highly effective in reducing collagen and fibroblast markers in mammary tumors (Figs 3 and 4) that was consistent with its efficacy in ameliorating fibrosis in non-malignant disease models of the kidney, liver, lung, heart and retina [38, 39, 53, 54] as well as in carcinogen [55] and MMTV-PyMT mammary tumorigenesis [56]. The fibrotic changes in the mammary gland of NeuT/ATTAC+AP mice (Figs 2 and 4) were similar to those described for invasive ductal breast carcinomas in terms of collagen abundance in fibrotic foci and its association with tumor progression and poor survival [9].

The major transcriptional effects of DMHCA were linked to transrepression of a network of LXR-responsive genes, including PTGS2, S100A9, SPP1, CD14, CCL5 and ANXA1, which are overexpressed in MDSC [57–62] and in a significant proportion of breast cancers [63], where they denote poor survival [57]. Breast cancer cell lines MDA-MB-231 and MDA-MB-435 are known to secrete CCL5 to increase the development of MDSC [64] and metastasis [65]. In a similar context, PTGS2 and its product PGE2 increase the differentiation of MDSC [66, 67] and their capacity to generate Treg cells [68]. LXR agonists were previously found to inhibit MDSC through the upregulation of ApoE and binding to the low density lipoprotein receptor LRP8 [69, 70], and the increase in ApoC1 transcription by DMHCA may have a similar function. Conversely, DMHCA increased expression of several LXR target genes, including SCD2, SREBF1, CPD and ABCA1 (S4 Table in S1 File), which positively correlate with increased survival in breast cancer subjects (Table 2).

Associated with reduction of tumor progression and fibrosis by DMHCA were its inhibitory effects on MDSC infiltration coincident and an increased percentage of CD4 and CD8 effector T cells. The latter changes occurred concurrently with a reduction in both naïve T cell populations in tumor infiltrates, but not in the spleen suggesting their differentiation in peripheral tissues [71]. This reduction in immune tolerance denoted in interactive LXR-downregulated mechanism (Fig 2C) that may have contributed to increased survival, and suggests that DMHCA may have further therapeutic potential in combination with immune checkpoint inhibitors.

Overall, the present study suggests that the pleiotropic actions of DMHCA and other LXR agonists work collectively to reduce collagen deposition and fibrosis [25, 26], proliferation [72–75] and the immune tolerant TME. The present findings offer the first evidence of the effectiveness of an LXR agonist in a stringent transgenic model of breast cancer fibrosis, and suggests a rationale for a new therapeutic approach to enhance the efficacy of therapies for HER2^+^ breast cancer and other malignancies.

## Supporting information

S1 File (PDF)
