# Supplementary material for: Reduction of fibrosis and immune suppressive cells in ErbB2-dependent tumorigenesis by an LXR agonist
Source: PLoS One. 2021 Mar 29;16(3):e0248996. doi: 10.1371/journal.pone.0248996 (PMC8007044; doi:10.1371/journal.pone.0248996)
Supplement: S1 File — (PDF) [file pone.0248996.s001.pdf]

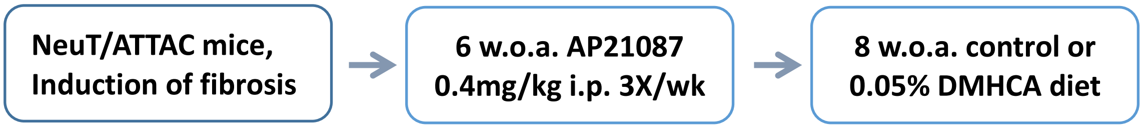

Figure S1. Schematic of the treatments of NeuT/ATTAC mice. See Methods for details. w.o.a., weeks of age

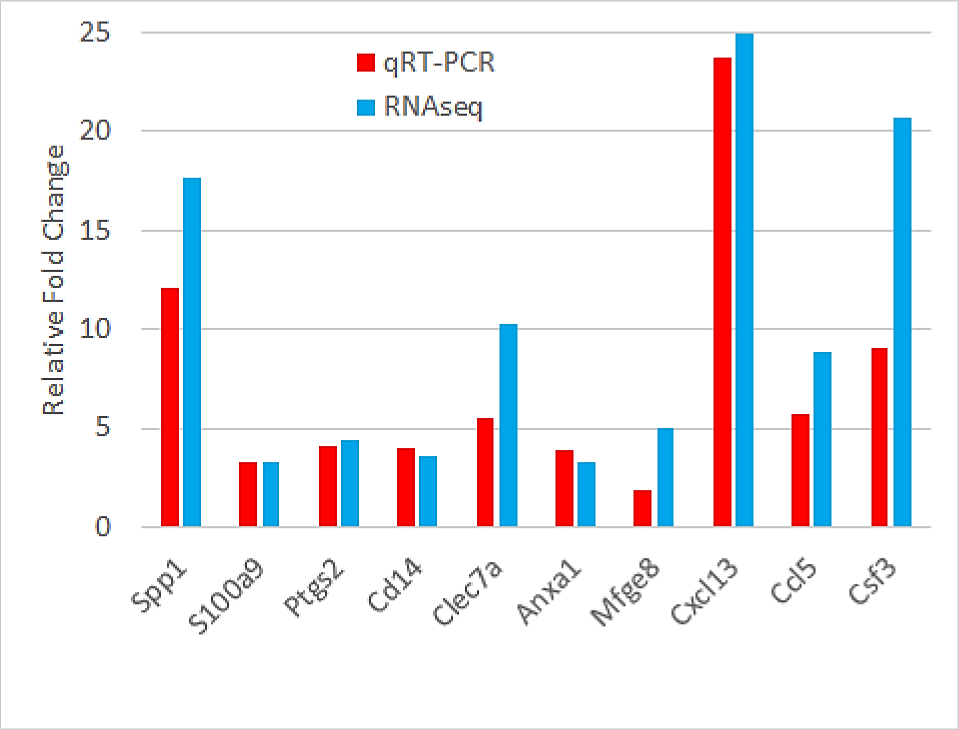

Figure S2. Comparison of qRT-PCR and RNAseq results of genes downregulated by DMHCA in mammary tumors from NeuT/ATTAC+AP mice. RNA selection is based on the genes in Fig 2C and RNAseq data in Table S3.

S1 Table. Antibodies for IHC and FACS

IHC:

**Antibody**	**Catalog #**	**Source**	**Dilution**
FAP	MBS176159	MyBiosource	1:120
S100A9	PA5-81470	Invitrogen	1:1000
CCL5	MA1-24665	Invitrogen	1:40
PD-1	AF1086	R&D	1:100
PD-L1	ab238697	Abcam	1:100
CXCL1	ab86436	Abcam	1:100
SPP1/Osteopontin	AF808	R&D	1:100

FACS:

**Antibody**	**Catalog #**	**Source**	**Conjugate**	**Clone**
Ly6G	127628	Biolegend	BV 421	1A8
CD11B	563168	BD	BV 711	M1/70
F4/80	123141	Biolegend	BV 785	BM8
Ly6C	553104	BD	FITC	AL-21
CD8A	100732	Biolegend	PerCPCy5.5	53-6.7
CD86	105008	Biolegend	PE	GL-1
MHC-II, I-A/I-E	25-5321-82	eBioscience	PE-Cy7	M5/114.15.2
CD80	102716	Biolegend	A647	16-10A
CD11C	565872	BD	APC-R700	N418
CD45	103116	Biolegend	APC-Cy7	30-F11
CD49B	563063	BD	BV 421	DX5
CD335	562850	BD	BV 421	29A14
CD25	740714	BD	BV 711	PC61
CD44	563736	BD	BV 785	IM7
CD4	553729	BD	FITC	GK1.5
CD3E	551163	BD	PerCPCy5.5	145-2C11
TCRB	560657	BD	PerCPCy5.5	H57-597
CD62L	553151	BD	PE	MEL-14
PD-1	109110	Biolegend	PE-Cy7	RMP1-30
FOXP3	17-5773-82	eBioscience	APC	FJK-16s
CD8	564983	BD	APC-R700	53-6.7
CD45	103116	Biolegend	APC-Cy7	30-F11
Live/Dead stain	564406	BD	BV 510	

S2 Table. List of primers for qRT-PCR analysis

Gene	Forward Primer (5'→3')	Reverse Primer (5'→3')
Lep	GGT TGA TCT CAC AAT GCG TTT C	TGG GAG ACA GGG TTC TAC TT
CD28	AGA GAC CCG GTG TAC CTT ATA G	GTG CAT ACC TGC CCT AAG AAA
Ptgs2	CGG ACT GGA TTC TAT GGT GAA A	CTT GAA GTG GGT CAG GAT GTA G
Tnfrsf13c	TGC CTT CAG ATG GTT GGA TAG	CAT ACC TCC AGC CCA GTA ATC
Ccl19	GGA AAC CAA GGA CCA GAA GAG	CTC TCT TGT CCA CAC TCA CAT C
Cxcl13	CTC GTG CCA AAT GGT TAC AAA G	CTT CAG GCA GCT CTT CTC TTA C
S100a9	CTG GGC TTA CAC TGC TCT TAC	GGT GTC GAT GAT GGT GGT TAT
Pou2F1	GTC TGC TTA TTT CTG CCT CTC T	GAT CGG CTT CAC CTG TTT CT
Spp1	CTT TCA CTC CAA TCG TCC CTA C	CAG AAA CCT GGA AAC TCC TAG AC
Ccl5	GCC CAC GTC AAG GAG TAT TT	CTT GAA CCC ACT TCT TCT CTG G
Cd14	TCA GTT ACA ACA GGC TGG ATA G	GGT GAC TAC GCC AGA GTT AAA

S3 Table. RNAseq analysis of tumors of NeuT/ATTAC+AP mice treated with DMHCA. Shown are genes with a raw score ≥300 in control or DMHCA-treated mice, with ≥ 3-fold change in expression between experimental groups and a p-value <0.05. Genes in **bold** contain an LXR response element (Pehkonen et al. BMC Genomics 13:50, 2012).

gene symbol	FC	p-value	padj	Control 1	Control 2	Control 3	Treated 1	Treated 2	Treated 3	Ctl Mean	DMHCA Mean
Ajap1	461.6	1.56E-06	3.63E-04	1	1	4	1,133	3	1,203	**2**	**780**
Gabra4	38.0	1.74E-02	1.87E-01	22	1	34	956	3	1,133	**19**	**697**
Tox3	8.8	2.48E-03	6.14E-02	50	25	54	527	7	601	**40**	**378**
Adra2c	13.0	2.37E-02	2.22E-01	178	3	22	1,197	55	1,369	**68**	**873**
Car6	10.8	1.11E-02	1.44E-01	8,373	744	2,652	60,688	2,404	63,553	**3,923**	**42,215**
Arg1	10.3	2.82E-04	1.54E-02	2,238	336	1,894	21,809	1,027	23,318	**1,489**	**15,384**
Trim9	10.2	5.31E-05	4.58E-03	126	16	31	275	1,206	280	**58**	**587**
Pde1c	9.4	1.93E-05	2.36E-03	351	29	85	1,614	954	1,802	**155**	**1,457**
Shisa7	9.1	4.30E-09	2.82E-06	147	42	83	992	460	1,010	**91**	**821**
Dpp10	8.6	7.92E-04	3.03E-02	3,114	86	435	10,929	8,300	12,006	**1,212**	**10,412**
Dppa1	8.4	1.15E-05	1.65E-03	88	10	56	490	236	545	**51**	**424**
Rec114	7.6	2.24E-08	1.14E-05	27	60	89	472	275	585	**59**	**444**
**Scd/Scd1**	7.4	5.15E-12	1.31E-08	36,642	27,311	72,810	329,193	337,700	341,092	**45,587**	**335,995**
**Apoc1**	7.0	1.75E-03	4.94E-02	1,256	237	3,229	15,383	1,724	16,135	**1,574**	**11,081**
Foxred2	7.0	8.07E-04	3.06E-02	102	31	237	1,192	143	1,254	**124**	**863**
Tlcd2	6.7	1.16E-03	3.81E-02	65	70	173	970	58	1,032	**102**	**687**
Nexmif	6.7	1.01E-02	1.37E-01	119	8	90	704	22	718	**72**	**481**
Slc26a9	6.2	1.23E-08	7.30E-06	220	156	67	830	1,021	887	**148**	**912**
B4galnt4	6.2	4.82E-04	2.18E-02	230	69	114	1,236	140	1,155	**137**	**844**
Rragd	6.0	4.09E-05	3.72E-03	287	138	340	2,030	408	2,123	**255**	**1,520**
Gldc	6.0	1.68E-05	2.17E-03	618	325	291	3,112	729	3,513	**412**	**2,451**
Susd4	5.6	1.36E-03	4.24E-02	186	13	88	636	287	679	**96**	**534**
Efhd1	5.6	7.41E-06	1.24E-03	217	69	344	1,014	1,423	1,079	**210**	**1,172**
Nrg1	5.4	6.73E-04	2.71E-02	401	50	297	1,693	564	1,742	**249**	**1,333**
Mfsd4a	5.3	3.51E-04	1.75E-02	2,161	670	2,978	13,861	3,011	14,117	**1,936**	**10,330**
Lrrc26	5.2	1.36E-05	1.87E-03	781	203	446	2,880	1,399	3,153	**477**	**2,477**
Olfr1388	5.1	9.30E-04	3.35E-02	186	983	145	2,612	1,308	2,805	**438**	**2,242**
Gdf5	5.0	9.56E-03	1.33E-01	131	33	427	1,395	179	1,364	**197**	**979**
Osbpl6	5.0	2.85E-03	6.58E-02	413	33	126	624	1,525	696	**191**	**948**
Sgsm1	5.0	1.52E-02	1.73E-01	129	48	103	675	17	690	**93**	**461**
Pcdhgc3	4.9	6.46E-03	1.06E-01	2,543	1,341	685	1,999	18,320	2,049	**1,523**	**7,456**
**Cdh23**	4.8	3.32E-03	7.16E-02	300	141	43	352	1,572	394	**161**	**773**
Aldh3b2	4.7	7.54E-04	2.95E-02	509	153	408	2,192	448	2,362	**357**	**1,667**
Ebf2	4.7	9.92E-03	1.36E-01	246	97	134	166	1,862	191	**159**	**740**
**Scd2**	4.4	8.26E-08	3.37E-05	31,374	21,698	11,861	93,248	97,898	96,720	**21,644**	**95,956**
Krt23	4.4	3.35E-04	1.70E-02	1,306	511	306	2,213	4,873	2,187	**708**	**3,091**
**Srebf1**	4.3	3.09E-10	3.35E-07	29,463	17,355	24,745	113,680	78,035	116,269	**23,854**	**102,661**
Deptor	4.1	1.19E-04	8.20E-03	13,598	4,054	4,410	34,061	21,282	36,068	**7,354**	**30,470**
Cyp24a1	4.1	6.81E-03	1.10E-01	194	14	190	593	475	573	**133**	**547**
Mest	4.1	7.83E-04	3.00E-02	131	222	154	327	1,378	363	**169**	**689**
Egf	4.1	1.36E-02	1.62E-01	103	47	210	636	64	761	**120**	**487**
Secisbp2l	4.1	1.41E-04	9.23E-03	20,460	8,980	15,382	77,862	23,978	80,104	**14,941**	**60,648**
Spdef	4.0	1.98E-02	2.00E-01	522	61	169	1,430	160	1,435	**251**	**1,008**
Padi2	4.0	1.77E-02	1.88E-01	1,037	477	2,245	7,158	506	7,347	**1,253**	**5,004**
Hal	4.0	1.17E-02	1.49E-01	640	336	655	622	5,262	608	**544**	**2,164**
Macroh2a2	4.0	1.07E-02	1.42E-01	463	126	144	356	2,170	393	**245**	**973**
Pycr1	3.9	2.08E-03	5.48E-02	801	717	488	3,527	548	3,776	**669**	**2,617**
Ldlrad3	3.9	1.35E-05	1.87E-03	786	261	406	1,985	1,630	2,016	**484**	**1,877**
Gpr161	3.8	8.46E-08	3.40E-05	204	99	142	555	535	622	**149**	**570**
Tgif2	3.8	9.11E-03	1.30E-01	367	84	237	1,197	177	1,254	**230**	**876**
Arfgef2	3.8	5.13E-04	2.28E-02	3,190	1,904	2,247	4,954	17,540	5,168	**2,447**	**9,221**
Ticrr	3.7	6.81E-05	5.51E-03	355	133	131	757	657	885	**206**	**766**
Gpc3	3.7	8.39E-03	1.24E-01	265	844	650	851	4,686	936	**586**	**2,158**
Asic1	3.7	6.25E-03	1.05E-01	298	98	308	1,185	207	1,192	**235**	**861**
Atp13a2	3.6	7.59E-06	1.25E-03	2,773	1,790	2,757	10,808	4,837	11,033	**2,440**	**8,893**
Wdr62	3.6	2.17E-06	4.60E-04	291	124	212	815	650	814	**209**	**760**
Eef1akmt3	3.6	1.66E-03	4.78E-02	135	93	89	477	114	547	**106**	**379**
Col27a1	3.6	2.62E-03	6.33E-02	3,710	1,152	844	8,858	3,650	7,894	**1,902**	**6,801**
Slc12a2	3.6	1.48E-05	1.98E-03	29,224	10,311	24,002	73,651	75,956	76,297	**21,179**	**75,301**
Plcb4	3.5	2.15E-04	1.24E-02	2,441	1,898	954	4,450	9,871	4,419	**1,764**	**6,246**
Tmem189	3.5	4.51E-04	2.07E-02	4,683	5,353	3,430	9,189	29,180	9,273	**4,489**	**15,881**
St3gal5	3.5	7.45E-08	3.09E-05	3,761	2,575	1,879	9,331	10,096	9,592	**2,739**	**9,673**
Irgq	3.5	1.42E-06	3.37E-04	4,404	1,861	2,888	10,677	10,448	11,156	**3,051**	**10,760**
Tspan1	3.5	5.32E-03	9.46E-02	4,481	1,261	733	8,792	4,567	9,462	**2,158**	**7,607**
Sox12	3.5	1.85E-03	5.04E-02	541	89	324	1,114	1,096	1,143	**318**	**1,118**
Kctd15	3.5	3.47E-05	3.39E-03	1,606	802	570	3,505	3,146	3,693	**992**	**3,448**
Apol9a	3.5	7.01E-03	1.11E-01	4,519	1,044	3,199	13,560	3,071	13,742	**2,921**	**10,124**
**Stil**	3.5	1.09E-06	2.72E-04	263	296	231	1,052	565	1,111	**263**	**910**
Apln	3.5	1.60E-09	1.21E-06	336	215	303	920	1,065	958	**284**	**981**

Zswim5	3.5	3.13E-02	2.55E-01	172	52	242	803	65	738	**155**	**535**
Reep6	3.4	2.76E-03	6.50E-02	177	55	167	560	194	615	**133**	**456**
Apol9b	3.4	8.01E-03	1.21E-01	2,615	699	2,067	8,366	1,647	8,419	**1,794**	**6,144**
Ccn3	3.4	4.59E-05	4.10E-03	406	858	430	2,165	1,265	2,364	**564**	**1,931**
Spata2	3.4	7.87E-03	1.19E-01	1,126	987	829	1,323	7,296	1,330	**981**	**3,316**
Pcdhgb6	3.3	1.18E-02	1.49E-01	508	489	170	553	2,735	618	**389**	**1,302**
Epop	3.3	3.99E-04	1.90E-02	342	286	436	1,505	486	1,557	**355**	**1,183**
Itpripl1	3.3	1.87E-06	4.16E-04	1,596	793	1,111	3,345	4,763	3,544	**1,167**	**3,884**
**Eef2k**	3.3	7.39E-04	2.90E-02	1,588	573	2,031	5,360	2,963	5,624	**1,397**	**4,649**
Car12	3.3	4.44E-02	3.02E-01	5,983	7,831	7,466	34,088	1,133	35,499	**7,093**	**23,573**
Ncaph	3.3	1.87E-04	1.11E-02	1,691	1,871	683	3,731	6,417	3,933	**1,415**	**4,694**
Zfp385b	3.3	1.91E-04	1.13E-02	1,229	835	408	2,976	1,978	3,141	**824**	**2,698**
Sbk1	3.3	2.82E-05	3.05E-03	1,903	720	1,357	3,939	4,872	4,204	**1,327**	**4,338**
Tnfrsf19	3.2	2.96E-03	6.71E-02	706	1,598	298	3,076	2,236	3,140	**867**	**2,817**
Stard13	3.2	9.50E-05	6.86E-03	1,113	484	425	2,163	2,241	2,147	**674**	**2,184**
Bhlha15	3.2	1.41E-05	1.92E-03	3,101	2,510	1,554	8,632	5,290	9,289	**2,388**	**7,737**
Sidt1	3.2	3.84E-05	3.58E-03	2,771	1,269	3,408	7,986	8,348	7,690	**2,483**	**8,008**
Adora1	3.2	1.10E-03	3.72E-02	1,267	1,082	917	4,658	1,227	4,636	**1,088**	**3,507**
**Lhpp**	3.2	3.47E-05	3.39E-03	402	210	417	1,233	756	1,306	**343**	**1,098**
Tox2	3.2	2.00E-02	2.01E-01	525	965	373	942	4,398	618	**621**	**1,986**
Eng	3.2	1.31E-03	4.13E-02	1,508	1,775	1,471	6,387	1,706	7,087	**1,584**	**5,060**
**Bckdha**	3.2	6.94E-06	1.21E-03	6,224	2,971	3,790	14,811	11,181	15,470	**4,328**	**13,821**
Ube2v1	3.2	2.79E-03	6.54E-02	6,795	6,708	4,881	10,141	38,076	10,306	**6,128**	**19,508**
Tyro3	3.2	1.19E-03	3.90E-02	1,240	479	989	3,547	1,363	3,679	**903**	**2,863**
Bckdk	3.2	2.86E-04	1.54E-02	3,364	1,972	2,758	10,460	4,031	11,156	**2,698**	**8,549**
Ncald	3.1	4.89E-04	2.20E-02	11,659	8,432	5,434	32,340	13,253	34,562	**8,508**	**26,718**
Aurka	3.1	6.50E-03	1.07E-01	1,108	1,910	600	2,147	6,852	2,190	**1,206**	**3,730**
Arhgef19	3.1	1.76E-04	1.07E-02	2,033	1,641	1,722	6,992	2,626	7,050	**1,799**	**5,556**
**Cpd**	3.1	2.23E-04	1.28E-02	36,920	17,826	28,908	102,210	48,262	107,160	**27,885**	**85,878**
Frmpd1	3.1	8.94E-04	3.27E-02	267	166	207	856	287	809	**213**	**651**
Plk1	3.0	1.18E-04	8.20E-03	1,199	1,277	499	3,102	2,654	3,287	**991**	**3,015**
Adrb1	3.0	2.41E-04	1.37E-02	2,016	1,079	927	4,797	2,569	4,857	**1,341**	**4,074**
Rcor2	3.0	3.08E-03	6.86E-02	180	260	85	651	266	673	**175**	**530**
B3gnt3	3.0	4.28E-02	2.97E-01	214	705	250	1,540	164	1,813	**390**	**1,173**
Adnp	3.0	1.80E-03	4.99E-02	1,753	1,380	1,490	2,903	8,476	2,512	**1,541**	**4,631**
Deaf1	3.0	6.66E-04	2.69E-02	1,402	765	1,333	4,286	1,703	4,528	**1,167**	**3,505**
Rassf10	3.0	2.39E-03	6.02E-02	1,457	411	646	2,916	1,649	2,959	**838**	**2,508**
Ggt6	3.0	3.09E-03	6.87E-02	140	98	143	511	139	489	**127**	**380**
Mpped2	3.0	1.32E-04	8.88E-03	439	275	184	939	694	1,053	**299**	**895**
**Acpp**	3.0	7.65E-02	3.94E-01	1,114	118	380	2,284	218	2,277	**537**	**1,593**
Mocs3	3.0	1.07E-02	1.42E-01	529	545	444	691	3,123	678	**506**	**1,497**
**Sox13**	3.0	9.32E-06	1.46E-03	1,849	1,084	936	3,977	3,454	3,993	**1,289**	**3,808**
Lrfn1	3.0	6.79E-03	1.09E-01	357	67	156	513	655	543	**193**	**570**
**Fam20c**	-3.0	2.37E-02	2.22E-01	269	187	1,313	196	190	214	**590**	**200**
Hc	-3.0	2.45E-02	2.26E-01	345	130	1,103	152	242	137	**526**	**177**
Dcn	-3.0	2.79E-02	2.42E-01	7,183	4,505	33,401	6,063	2,637	6,397	**15,030**	**5,032**
Ptger4	-3.0	4.93E-03	9.10E-02	473	370	1,347	291	154	287	**730**	**244**
Gvin1	-3.0	1.03E-02	1.39E-01	4,385	1,173	8,686	1,477	1,722	1,545	**4,748**	**1,581**
Wnt7b	-3.1	9.69E-03	1.35E-01	1,200	271	528	132	365	152	**670**	**216**
Pik3r5	-3.1	3.87E-03	7.88E-02	349	363	1,288	230	206	220	**667**	**218**
Pir	-3.1	7.24E-03	1.13E-01	864	1,175	182	240	251	229	**740**	**240**
Vmn1r229	-3.1	7.44E-03	1.15E-01	682	270	1,686	299	238	314	**879**	**284**
Wnt5a	-3.1	2.86E-03	6.59E-02	2,718	1,658	608	433	729	437	**1,660**	**533**
Wnt5b	-3.1	1.12E-02	1.46E-01	1,630	390	1,921	508	173	578	**1,780**	**420**
Tnfrsf13b	-3.1	1.05E-02	1.41E-01	218	134	792	116	110	140	**381**	**122**
Rac2	-3.2	1.33E-02	1.60E-01	1,215	825	5,722	805	800	852	**2,588**	**819**
H2-DMb2	-3.2	3.16E-04	1.63E-02	1,299	1,874	3,983	804	650	811	**2,385**	**755**
Dram1	-3.2	2.23E-05	2.56E-03	674	875	1,553	317	326	338	**1,034**	**327**
Ptprc	-3.2	9.22E-03	1.31E-01	3,587	1,942	13,117	2,051	1,807	2,030	**6,215**	**1,963**
Myo1g	-3.2	2.70E-02	2.38E-01	480	247	2,443	385	220	395	**1,057**	**333**
**Slpi**	-3.2	2.75E-03	6.48E-02	1,027	634	2,013	481	179	495	**1,224**	**385**
Ccdc141	-3.2	6.17E-03	1.04E-01	786	180	864	231	109	235	**610**	**192**
Pdgfd	-3.2	2.08E-05	2.45E-03	1,445	2,715	1,302	627	449	633	**1,821**	**570**
St3gal6	-3.2	1.86E-08	1.03E-05	450	642	674	183	185	185	**589**	**184**
Mtmr11	-3.2	8.26E-05	6.23E-03	456	1,039	482	211	174	231	**659**	**205**
H2-Ab1	-3.2	1.03E-05	1.56E-03	13,941	17,059	31,326	6,754	5,843	6,782	**20,775**	**6,460**
**Lpl**	-3.2	1.41E-02	1.66E-01	3,634	3,144	20,936	3,051	2,530	3,028	**9,238**	**2,869**
Loxl4	-3.2	4.07E-02	2.92E-01	718	566	75	59	285	78	**453**	**141**
Lmcd1	-3.2	4.98E-02	3.20E-01	1,854	6,371	338	521	1,648	468	**2,854**	**879**
Aatk	-3.3	7.82E-03	1.19E-01	1,467	1,340	282	240	512	197	**1,030**	**316**
Col16a1	-3.3	3.16E-13	1.13E-09	12,269	9,784	11,969	3,665	3,252	3,431	**11,340**	**3,449**
Ablim1	-3.3	1.11E-03	3.72E-02	1,603	1,745	5,207	991	611	999	**2,852**	**867**
**Glipr1**	-3.3	1.86E-03	5.06E-02	377	245	962	200	121	162	**528**	**161**
Pde3b	-3.3	5.62E-03	9.82E-02	381	392	1,791	269	256	254	**855**	**259**

**Cd83**	-3.3	4.76E-03	8.93E-02	548	1,102	2,946	547	290	555	**1,532**	**464**
S100a9	-3.3	9.83E-04	3.46E-02	303	300	304	50	157	53	**302**	**87**
Steap3	-3.3	1.51E-03	4.53E-02	3,819	6,573	1,859	920	1,941	840	**4,084**	**1,233**
A2m	-3.3	4.64E-02	3.09E-01	2,488	8,498	357	1,511	495	1,415	**3,781**	**1,141**
**Anxa1**	-3.3	6.12E-07	1.73E-04	14,224	21,366	20,134	6,432	3,790	6,539	**18,575**	**5,587**
Ttll7	-3.3	3.99E-03	8.01E-02	321	836	717	255	61	247	**624**	**188**
Il16	-3.3	1.84E-02	1.93E-01	569	232	2,500	381	274	334	**1,100**	**330**
Ebf1	-3.4	1.02E-03	3.54E-02	705	674	2,309	332	416	348	**1,229**	**366**
Scn5a	-3.4	7.77E-04	3.00E-02	1,962	3,430	776	625	556	653	**2,056**	**611**
Agbl2	-3.4	4.57E-03	8.72E-02	342	491	96	72	136	66	**310**	**91**
Plscr2	-3.4	8.57E-14	3.56E-10	2,280	2,750	2,897	800	744	759	**2,642**	**768**
Krt7	-3.4	3.45E-04	1.73E-02	9,241	19,979	5,258	3,141	3,828	3,041	**11,493**	**3,337**
Il2rg	-3.5	1.97E-02	2.00E-01	641	338	3,764	466	378	529	**1,581**	**458**
H2-Aa	-3.5	2.01E-06	4.32E-04	19,226	25,582	44,375	8,469	8,585	8,626	**29,727**	**8,560**
**Coro1a**	-3.5	7.13E-03	1.12E-01	2,826	1,926	12,795	1,963	1,218	1,841	**5,849**	**1,674**
Galnt15	-3.5	3.15E-08	1.51E-05	1,592	1,271	1,599	488	294	493	**1,487**	**425**
Cyfip2	-3.5	1.73E-02	1.86E-01	306	110	1,352	171	169	165	**589**	**168**
Hspb8	-3.5	1.35E-03	4.21E-02	1,905	3,898	845	742	400	747	**2,216**	**630**
Rbp7	-3.5	3.60E-02	2.74E-01	5,362	33,016	2,540	2,315	7,393	1,913	**13,639**	**3,874**
Dclk1	-3.6	6.64E-07	1.81E-04	1,114	1,369	1,023	228	465	290	**1,169**	**328**
Bhlhe41	-3.6	2.94E-04	1.56E-02	4,912	5,817	8,633	2,377	712	2,332	**6,454**	**1,807**
**Il1rn**	-3.6	2.60E-09	1.80E-06	626	456	639	147	190	146	**574**	**161**
Wwp2	-3.6	4.93E-04	2.22E-02	9,468	21,478	4,861	3,162	3,716	3,136	**11,936**	**3,338**
Map1b	-3.6	5.36E-05	4.59E-03	1,057	2,425	2,371	646	335	656	**1,951**	**546**
Serpinb5	-3.6	3.78E-03	7.77E-02	2,679	5,063	4,657	1,584	256	1,619	**4,133**	**1,153**
Cd14	-3.6	1.50E-07	5.34E-05	16,631	11,140	23,511	4,789	4,426	5,059	**17,094**	**226**
Ifi211	-3.6	2.33E-03	5.92E-02	338	540	1,560	246	135	297	**813**	**226**
Gbp4	-3.6	4.13E-02	2.93E-01	793	176	4,659	670	236	642	**1,876**	**516**
**Egr2**	-3.7	1.16E-02	1.48E-01	1,038	1,992	481	137	693	131	**1,170**	**320**
Hba-a2	-3.7	6.88E-06	1.21E-03	8,348	17,712	21,818	4,923	3,591	4,581	**15,959**	**4,365**
Pamr1	-3.7	1.45E-03	4.40E-02	311	890	1,042	260	88	266	**748**	**205**
Lef1	-3.7	1.62E-02	1.79E-01	621	51	854	118	156	144	**509**	**139**
As3mt	-3.7	6.43E-06	1.15E-03	859	1,528	1,181	398	190	387	**1,189**	**325**
Gcnt4	-3.7	4.09E-04	1.93E-02	472	579	156	95	142	91	**402**	**109**
**Sash3**	-3.7	9.94E-03	1.36E-01	484	240	2,357	327	228	272	**1,027**	**276**
Ggt5	-3.7	5.74E-03	9.98E-02	409	291	1,943	248	176	285	**881**	**236**
Fam49a	-3.7	8.17E-04	3.08E-02	593	437	1,827	225	322	218	**952**	**255**
Fads3	-3.8	1.42E-03	4.35E-02	795	3,132	1,796	634	216	673	**1,908**	**507**
Mmp12	-3.8	1.42E-02	1.66E-01	470	666	4,512	651	296	545	**1,883**	**497**
Muc15	-3.8	1.36E-02	1.62E-01	14,971	4,357	42,725	7,872	1,734	6,766	**20,685**	**5,457**
**Mical1**	-3.8	3.41E-05	3.36E-03	632	2,014	1,216	387	273	354	**1,287**	**338**
Eva1a	-3.8	1.85E-02	1.94E-01	361	1,623	97	134	259	150	**693**	**181**
Rbp1	-3.8	3.85E-04	1.86E-02	21,434	51,880	11,912	8,608	5,283	8,285	**28,409**	**7,392**
Anxa3	-3.9	3.05E-03	6.82E-02	11,995	29,998	6,166	2,600	7,499	2,362	**16,053**	**4,153**
Clec7a	-3.9	3.41E-04	1.72E-02	342	451	1,181	128	236	147	**658**	**171**
Ptges	-3.9	3.30E-03	7.14E-02	563	256	1,711	220	136	298	**843**	**218**
Pkp1	-3.9	1.05E-03	3.61E-02	9,511	5,872	2,530	1,957	602	2,022	**5,971**	**1,527**
Arhgap45	-3.9	6.38E-03	1.06E-01	1,468	665	6,321	871	501	786	**2,818**	**719**
Syt12	-3.9	2.93E-04	1.56E-02	350	981	303	119	192	107	**545**	**139**
Bace2	-3.9	5.32E-04	2.32E-02	2,094	3,487	958	445	901	321	**2,179**	**556**
C3	-3.9	9.43E-03	1.32E-01	3,530	1,782	18,747	2,463	1,271	2,386	**8,020**	**2,040**
Nt5e	-3.9	3.30E-02	2.63E-01	1,647	9,820	1,511	349	2,647	297	**4,326**	**1,098**
Nkd2	-3.9	7.99E-03	1.20E-01	4,202	7,311	736	688	1,835	580	**4,083**	**1,034**
Ccdc68	-4.0	4.02E-05	3.68E-03	597	895	304	180	102	173	**599**	**152**
Sparcl1	-4.0	2.80E-02	2.42E-01	17,302	122,141	10,119	5,894	26,322	5,500	**49,854**	**12,572**
Mmp3	-4.0	4.85E-07	1.44E-04	1,884	2,195	3,958	722	510	786	**2,679**	**672**
Arhgap6	-4.0	7.79E-10	6.69E-07	788	807	638	154	244	157	**744**	**185**
Fth1	-4.1	2.96E-05	3.11E-03	102,984	247,898	71,765	37,126	31,026	35,204	**140,882**	**34,452**
Tns4	-4.1	1.81E-03	4.99E-02	1,279	3,163	368	382	375	410	**1,603**	**389**
Alkal2	-4.2	5.23E-03	9.36E-02	16,070	22,307	2,728	2,419	5,957	1,505	**13,701**	**3,294**
Cdo1	-4.2	3.90E-03	7.90E-02	830	409	2,627	401	114	407	**1,289**	**307**
Clu	-4.2	3.55E-05	3.44E-03	61,229	50,394	40,402	7,420	21,660	7,122	**50,675**	**12,067**
Sec14l5	-4.3	2.53E-02	2.29E-01	924	676	30	69	242	70	**543**	**127**
Map6	-4.3	4.25E-04	1.99E-02	782	1,466	246	199	226	157	**831**	**194**
Ptn	-4.3	3.77E-03	7.77E-02	1,894	948	8,683	1,066	559	1,040	**3,842**	**889**
Ttyh2	-4.3	3.81E-03	7.80E-02	509	3,091	486	245	482	217	**1,362**	**315**
Gimap6	-4.3	3.68E-03	7.62E-02	654	503	4,101	392	386	436	**1,753**	**405**
Car3	-4.4	4.42E-04	2.04E-02	7,343	6,307	24,618	3,589	1,509	3,688	**12,756**	**2,929**
Chrnb1	-4.4	5.84E-10	5.60E-07	2,720	3,813	1,897	682	629	620	**2,810**	**643**
Ptgs2	-4.4	7.61E-04	2.95E-02	625	160	1,122	161	105	166	**635**	**144**
Arxes2	-4.5	3.55E-06	6.81E-04	278	326	665	92	122	67	**423**	**94**
Rasd1	-4.5	5.24E-12	1.31E-08	426	363	522	85	93	112	**437**	**97**
Syn3	-4.6	1.02E-03	3.55E-02	271	1,141	809	214	51	222	**740**	**162**
Cyp4f13	-4.6	1.81E-06	4.11E-04	998	1,158	1,046	342	105	255	**1,067**	**234**

Grap	-4.6	5.13E-03	9.29E-02	259	127	1,516	145	131	141	**634**	**139**
Adam33	-4.6	8.65E-11	1.13E-07	468	457	296	89	98	79	**407**	**89**
Lamb3	-4.6	1.06E-02	1.41E-01	1,661	539	4,891	769	80	688	**2,364**	**513**
Hvcn1	-4.6	1.30E-03	4.11E-02	790	444	3,409	333	274	398	**1,548**	**335**
Mgam	-4.6	2.62E-03	6.33E-02	650	2,038	440	105	457	115	**1,042**	**226**
Alcam	-4.6	9.46E-07	2.43E-04	1,150	527	1,604	249	205	254	**1,094**	**236**
Cp	-4.8	3.74E-08	1.73E-05	39,886	29,022	66,916	8,698	12,286	7,594	**45,275**	**9,526**
Vtcn1	-4.8	3.20E-03	7.01E-02	3,321	3,532	1,041	194	1,254	200	**2,631**	**549**
Kcne3	-4.8	4.23E-03	8.29E-02	759	3,417	224	256	388	273	**1,467**	**306**
Hmgn3	-4.8	1.51E-05	2.00E-03	965	3,264	1,213	306	501	324	**1,814**	**377**
Chadl	-4.9	2.26E-02	2.17E-01	1,552	5,047	177	167	1,067	154	**2,259**	**463**
Sspn	-4.9	2.24E-07	7.35E-05	998	2,170	799	274	284	254	**1,323**	**271**
**Cd52**	-4.9	5.51E-04	2.38E-02	1,012	889	5,202	564	374	499	**2,368**	**479**
**Plb1**	-5.0	1.64E-04	1.02E-02	694	371	1,966	284	166	159	**1,010**	**203**
Mfge8	-5.0	5.19E-12	1.31E-08	200,063	113,525	173,415	33,528	38,303	26,198	**162,334**	**32,676**
Ripor2	-5.1	4.76E-05	4.19E-03	487	885	1,588	117	332	137	**987**	**195**
**Gimap4**	-5.1	2.46E-03	6.12E-02	686	408	4,441	340	429	325	**1,845**	**364**
Spink5	-5.1	2.74E-02	2.39E-01	353	77	1,240	168	6	155	**557**	**110**
Pard3b	-5.2	8.61E-12	1.95E-08	306	390	410	82	52	80	**368**	**72**
Mill2	-5.2	3.11E-04	1.62E-02	1,308	529	1,073	125	374	61	**970**	**187**
Moxd1	-5.5	9.65E-06	1.50E-03	5,735	5,712	3,263	1,260	294	1,139	**4,903**	**898**
Sdcbp2	-5.5	3.86E-07	1.17E-04	530	1,384	534	115	185	144	**816**	**148**
Areg	-5.6	4.67E-05	4.14E-03	213	868	773	133	52	146	**618**	**110**
Ptprz1	-5.6	1.24E-04	8.49E-03	333	97	655	58	83	52	**362**	**65**
Aspa	-5.7	1.25E-04	8.54E-03	319	1,248	227	126	72	118	**598**	**106**
Angpt1	-5.7	1.66E-04	1.02E-02	867	186	1,707	163	163	158	**920**	**162**
Aldh1a3	-5.7	9.55E-07	2.43E-04	4,787	2,222	2,163	327	874	410	**3,057**	**537**
**Fabp4**	-5.7	8.25E-05	6.23E-03	4,893	4,360	23,974	1,996	1,777	2,013	**11,076**	**1,928**
S100a6	-5.8	7.09E-10	6.54E-07	11,374	24,231	10,279	2,380	3,030	2,447	**15,295**	**2,619**
Kbtbd11	-5.9	3.73E-04	1.82E-02	513	283	2,290	204	116	208	**1,029**	**176**
Klk10	-5.9	7.32E-03	1.14E-01	320	3,877	1,120	103	723	76	**1,772**	**301**
Samd12	-6.0	2.70E-04	1.49E-02	420	878	250	57	175	30	**516**	**87**
Cryab	-5.9	2.44E-05	2.73E-03	2,503	10,078	2,165	981	605	899	**4,915**	**828**
Il18r1	-6.0	9.30E-05	6.78E-03	2,318	2,337	2,360	157	863	152	**2,338**	**391**
Ptpn7	-6.1	7.56E-05	5.94E-03	211	1,823	932	174	146	169	**989**	**163**
Ldhd	-6.2	3.18E-03	6.98E-02	24,521	81,507	3,190	3,679	10,805	3,063	**36,406**	**5,849**
Tcf7	-6.3	2.86E-03	6.59E-02	400	74	1,684	68	197	77	**719**	**114**
Grap2	-6.4	6.18E-04	2.56E-02	363	115	1,623	106	106	117	**701**	**109**
Cytip	-6.6	8.09E-07	2.14E-04	2,964	3,145	8,157	903	338	914	**4,755**	**718**
Ighm	-7.1	2.59E-04	1.44E-02	1,317	772	8,694	526	494	503	**3,594**	**508**
Cd22	-7.3	9.46E-04	3.38E-02	376	78	1,941	98	107	123	**798**	**109**
Cd37	-7.4	1.80E-03	4.99E-02	610	123	3,961	268	140	231	**1,565**	**213**
Wfdc2	-7.5	4.21E-11	6.17E-08	719	1,124	790	145	59	146	**877**	**117**
Itih2	-7.7	6.65E-02	3.67E-01	1,054	2,944	169	23	506	17	**1,389**	**182**
**Gimap8**	-7.7	8.69E-05	6.52E-03	692	233	2,866	164	158	172	**1,264**	**165**
Sema3d	-7.9	3.08E-02	2.54E-01	2,509	9,870	221	212	1,149	231	**4,200**	**531**
Dpyd	-8.0	1.58E-04	9.96E-03	339	50	530	62	18	37	**306**	**39**
S100a14	-8.1	1.73E-03	4.92E-02	458	2,699	199	58	316	43	**1,119**	**139**
Nat8f2	-8.1	2.19E-10	2.60E-07	422	450	753	99	66	36	**542**	**67**
Ror2	-8.2	9.52E-05	6.86E-03	500	1,586	129	69	141	63	**738**	**91**
**Ccl5**	-8.9	1.35E-03	4.21E-02	219	73	2,649	117	117	99	**980**	**111**
Plin4	-9.2	1.65E-08	9.56E-06	629	236	1,184	81	75	67	**683**	**74**
CD209b	-9.4	2.89E-05	3.06E-03	368	154	1,708	94	52	93	**743**	**80**
Sema3b	-9.6	1.67E-05	2.17E-03	1,304	4,906	357	265	202	221	**2,189**	**229**
Lyz1	-9.8	1.47E-03	4.45E-02	422	79	3,542	185	42	189	**1,348**	**138**
Vmn2r124	-10.8	7.39E-05	5.85E-03	489	69	1,527	88	32	74	**695**	**65**
Ecrg4	-10.9	1.24E-06	3.00E-04	930	7,973	1,870	260	455	273	**3,591**	**329**
Prss35	-10.9	2.58E-04	1.44E-02	2,167	1,540	146	45	275	34	**1,285**	**118**
Acap1	-11.0	1.74E-07	5.94E-05	711	548	2,703	88	193	82	**1,321**	**121**
Tnfrsf13c	-11.4	9.54E-04	3.40E-02	128	18	1,062	49	16	42	**403**	**36**
Cd3g	-11.4	1.85E-02	1.94E-01	208	21	2,193	85	37	91	**807**	**71**
**Scrn1**	-11.5	1.13E-02	1.47E-01	802	5,011	130	96	371	51	**1,981**	**173**
Slc44a4	-11.8	2.51E-10	2.84E-07	404	1,108	277	57	53	42	**596**	**51**
Krt19	-12.2	2.33E-02	2.20E-01	1,949	324	20,355	890	64	896	**7,543**	**617**
Hp	-13.8	5.14E-10	5.12E-07	1,228	1,150	5,308	204	169	186	**2,562**	**186**
Ly6d	-14.2	1.54E-05	2.03E-03	1,073	374	3,918	167	21	191	**1,788**	**126**
Ltb	-14.5	7.49E-05	5.91E-03	775	85	4,539	157	80	138	**1,800**	**125**
Otud7a	-15.2	3.58E-30	8.92E-26	810	680	1,121	66	51	56	**870**	**58**
Pinc	-15.4	6.68E-03	1.09E-01	2,737	100	11,049	409	97	397	**4,629**	**301**
Tnnt2	-15.6	4.38E-05	3.94E-03	1,107	9,679	347	147	439	128	**3,711**	**238**
**Spp1**	-15.7	2.67E-16	1.33E-12	506,819	857,468	509,233	26,917	67,452	24,617	**624,507**	**39,662**
Klrb1a	-16.4	1.16E-02	1.48E-01	396	1,977	36	19	123	7	**803**	**50**
Ltf	-17.4	3.60E-03	7.53E-02	27,341	3,090	231,883	6,285	2,416	6,371	**87,438**	**5,024**
Klk6	-18.7	5.63E-08	2.48E-05	67	1,004	484	26	27	32	**518**	**28**

Enpp2	-18.9	1.15E-11	2.05E-08	804	1,033	3,938	126	70	110	**1,925**	**102**
Smoc1	-22.5	1.92E-25	2.39E-21	3,561	2,487	5,988	227	129	181	**4,012**	**179**
Ighg2b	-22.5	9.71E-06	1.50E-03	360	13	982	28	14	20	**452**	**21**
Plin1	-23.0	2.29E-07	7.41E-05	530	87	1,487	31	51	11	**701**	**31**
Wfdc5	-23.1	7.19E-03	1.13E-01	3,564	13,480	71	71	611	60	**5,705**	**247**
Sftpd	-23.5	1.14E-11	2.05E-08	693	592	3,046	52	92	42	**1,444**	**62**
Kcna6	-24.1	2.45E-07	7.83E-05	1,313	2,099	164	25	109	16	**1,192**	**50**
Cxcl13	-24.9	1.53E-03	4.54E-02	237	64	4,117	53	83	42	**1,472**	**60**
Serpinb11	-25.3	1.21E-06	2.96E-04	312	2,565	396	75	5	51	**1,091**	**44**
Trpm6	-25.9	1.27E-24	1.05E-20	602	779	685	32	12	38	**688**	**27**
Csn1s1	-26.1	9.07E-03	1.30E-01	30,140	808	345,672	6,803	600	7,022	**125,540**	**4,808**
Trpv6	-28.0	1.13E-03	3.76E-02	708	2,376	56	16	90	8	**1,047**	**38**
Mup22	-28.6	2.43E-06	4.96E-04	471	79	4,976	75	57	63	**1,842**	**65**
Igkc	-29.5	6.04E-04	2.55E-02	1,846	119	12,891	209	174	122	**4,952**	**168**
**Cfd**	-31.6	4.24E-08	1.92E-05	2,523	387	8,969	92	236	48	**3,960**	**126**
Adipoq	-33.5	2.25E-11	3.54E-08	710	283	3,031	35	54	33	**1,341**	**40**
Cidec	-36.0	5.86E-11	8.11E-08	504	298	2,701	38	45	16	**1,168**	**33**
Dcpp1	-39.8	3.06E-04	1.60E-02	19,268	255	1,409	259	91	177	**6,977**	**176**
Csn1s2a	-41.5	3.56E-03	7.48E-02	20,062	527	341,902	4,190	539	4,001	**120,830**	**2,910**
Ighg1	-43.5	3.01E-04	1.59E-02	541	6	1,282	22	10	12	**610**	**15**
Sell	-48.8	1.79E-04	1.08E-02	401	23	3,373	23	32	24	**1,265**	**26**
Pck1	-59.2	9.30E-11	1.16E-07	344	55	1,144	12	11	5	**515**	**9**
Ighd	-57.6	3.15E-04	1.63E-02	739	16	5,949	52	28	38	**2,235**	**39**
Ms4a1	-77.6	3.03E-04	1.59E-02	475	4	3,663	22	13	20	**1,381**	**18**
Ccl19	-86.0	3.20E-05	3.27E-03	178	22	2393	14	7	11	**864**	**11**
Csn2	-108.0	1.59E-04	1.00E-02	28,038	765	515,126	2,400	682	1,954	**181,310**	**1,679**
Cd19	-111.0	9.04E-05	6.67E-03	343	6	3,191	19	8	7	**1,180**	**11**
Cd79a	-111.2	1.58E-04	9.94E-03	526	4	5,277	23	14	17	**1,936**	**18**
Bpifa2	-178.2	3.27E-04	1.67E-02	154,470	108	690	426	156	290	**51,756**	**291**
Amy1	-205.4	3.59E-05	3.47E-03	78,619	175	1,027	184	103	103	**26,607**	**130**
Glycam1	-213.9	1.79E-05	2.26E-03	2,022	19	21,178	61	30	19	**7,739**	**37**
Npy	-217.5	1.22E-05	1.73E-03	647	11	7,804	22	10	9	**2,821**	**13**
Cr2	-247.3	7.65E-06	1.25E-03	568	5	4,887	10	7	7	**1,820**	**8**
Chia1	-338.9	6.94E-05	5.60E-03	31,959	17	152	49	29	18	**10,709**	**32**
Crisp1	-350.6	1.41E-04	9.23E-03	2,080	2	11	4	2	2	**698**	**2**

S4 Table. Genes modulated by DMHCA that contain an LXR response element.
Tumors from NeuT/ATTAC+AP mice were analyzed by RNAseq. Shown are genes with a raw score >300 in either group, ≥3-fold change in expression and an LXR response element (Pehkonen et al. BMC Genomics 13:50, 2012). N=3 per group.

gene symbol	FC	pvalue	padj	Control Mean	DMHCA Mean
Scd1/Scd	7.4	5.15E-12	1.31E-08	45,587	335,995
Apoc1	7.0	1.75E-03	4.94E-02	1,574	11,081
Cdh23	4.8	3.32E-03	7.16E-02	161	773
Scd2/Scd5	4.4	8.26E-08	3.37E-05	21,644	95,956
Srebf1	4.3	3.09E-10	3.35E-07	23,854	102,661
Stil	3.5	1.09E-06	2.72E-04	263	910
Eef2k	3.3	7.39E-04	2.90E-02	1,397	4,649
Lhpp	3.2	3.47E-05	3.39E-03	343	1,098
Bckdha	3.2	6.94E-06	1.21E-03	4,328	13,821
Cpd	3.1	2.23E-04	1.28E-02	27,885	85,878
Acpp	3.0	7.65E-02	3.94E-01	537	1,593
Sox13	3.0	9.32E-06	1.46E-03	1,289	3,808
Abca1	2.8	2.08E-05	2.45E-03	4,862	13,428
Fam20c	0.34	2.37E-02	2.22E-01	590	200
Slpi	0.31	2.75E-03	6.48E-02	1,224	385
Lpl	0.31	1.41E-02	1.66E-01	9,238	2,869
Glipr1	0.30	1.86E-03	5.06E-02	528	161
Cd83	0.30	4.76E-03	8.93E-02	1,532	464
Anxa1	0.30	6.12E-07	1.73E-04	18,575	5,587
S100a9	0.29	9.83E-04	3.46E-02	302	87
Coro1a	0.29	7.13E-03	1.12E-01	5,849	1,674
Il1rn	0.28	2.60E-09	1.80E-06	574	161
Egr2	0.27	1.16E-02	1.48E-01	1,170	320
Sash3	0.27	9.94E-03	1.36E-01	1,027	276
Mical1	0.26	3.41E-05	3.36E-03	1,287	338
Ptges	0.26	3.30E-03	7.14E-02	843	218
Cd52	0.20	5.51E-04	2.38E-02	2,368	479
Plb1	0.20	1.64E-04	1.02E-02	1,010	203
Gimap4	0.20	2.46E-03	6.12E-02	1,845	364
Fabp4	0.17	8.25E-05	6.23E-03	11,076	1,928
Gimap8	0.13	8.69E-05	6.52E-03	1,264	165
Ccl5	0.11	1.35E-03	4.21E-02	980	111
Scrn1	0.09	1.13E-02	1.47E-01	1,981	173
Spp1	0.08	2.67E-16	1.33E-12	624,507	39,662
Cfd	0.03	4.24E-08	1.92E-05	3,960	126
